# Investigation of the structure-activity relationship at the *N*-terminal part of minigastrin analogs

**DOI:** 10.1186/s13550-023-01016-y

**Published:** 2023-07-08

**Authors:** Nadine Holzleitner, Thomas Günther, Amira Daoud-Gadieh, Constantin Lapa, Hans-Jürgen Wester

**Affiliations:** 1 Department of Chemistry, Technical University of Munich, 85748 Garching, Germany; 2 Nuclear Medicine, University Hospital Augsburg, 86156 Augsburg, Germany

**Keywords:** Cholecystokinin-2 receptor (CCK-2R), Cholecystokinin-B receptor (CCK-BR), Medullary thyroid carcinoma (MTC), Minigastrin, Tetrapeptide

## Abstract

**Background:**

Over the last years, several strategies have been reported to improve the metabolic stability of minigastrin analogs. However, currently applied compounds still reveal limited in vitro and in vivo stability. We thus performed a glycine scan at the *N*-terminus of DOTA-MGS5 (DOTA-d-Glu-Ala-Tyr-Gly-Trp-(*N*-Me)Nle-Asp-1-Nal) to systematically analyze the peptide structure. We substituted *N*-terminal amino acids by simple PEG spacers and investigated in vitro stability in human serum. Furthermore, we evaluated different modifications on its tetrapeptide binding sequence (*H*-Trp-(*N*-Me)Nle-Asp-1-Nal-NH_2_).

**Results:**

Affinity data of all glycine scan peptides were found to be in a low nanomolar range (4.2–8.5 nM). However, a truncated compound lacking the d-γ-Glu-Ala-Tyr sequence revealed a significant loss in CCK-2R affinity. Substitution of the d-γ-Glu-Ala-Tyr-Gly sequence of DOTA-γ-MGS5 (DOTA- d-γ-Glu-Ala-Tyr-Gly-Trp-(*N-*Me)Nle-Asp-1-Nal-NH_2_) by polyethylene glycol (PEG) spacers of different length exhibited only a minor influence on CCK-2R affinity and lipophilicity. However, in vitro stability of the PEG-containing compounds was significantly decreased. In addition, we confirmed that the tetrapeptide sequence *H*-Trp-Asp-(*N-*Me)Nle-1-Nal-NH_2_ is indeed sufficient for high CCK-2R affinity.

**Conclusion:**

We could demonstrate that a substitution of d-γ-Glu-Ala-Tyr-Gly by PEG spacers simplified the peptide structure of DOTA-MGS5 while high CCK-2R affinity and favorable lipophilicity were maintained. Nevertheless, further optimization with regard to metabolic stability must be carried out for these minigastrin analogs.

**Supplementary Information:**

The online version contains supplementary material available at 10.1186/s13550-023-01016-y.

## Introduction

In 1999, Behr et al*.* reported first studies on the human peptide hormone minigastrin (*H-*Leu-(GIu)_5_-Ala-Tyr-Gly-Trp-Met-Asp-Phe-NH_2_) and its diethylenetriaminepentaacetic acid (DTPA)-conjugated analog targeting the cholecystokinin-2 receptor (CCK-2R) [1], which is overexpressed in a high percentage on several human tumor types such as medullary thyroid carcinoma (MTC, 92%), small cell lung cancer (57%), stromal ovarian cancer (100%) and astrocytoma (65%) [2, 3]. Over the years, many modifications have been published, improving the pharmacokinetic properties of radiolabeled minigastrin derivatives. For example, the substitution of Leu by d-Glu led to an improved complex stability of [^111^In]In- and [^88^Y]Y-DTPA-MG0 (DTPA-d-Glu-(GIu)_5_-Ala-Tyr-Gly-Trp-Met-Asp Phe-NH_2_) [4]. Due to an observed elevated kidney accumulation, approaches to decrease this uptake by a reduction of the *N-*terminal D-glutamate chain as for MG11 (*H*-d-Glu-Ala-Tyr-Gly-Trp-Met-Asp-Phe-NH_2_) [5, 6], or the substitution of the six L- by six D-glutamate moieties as for CP04 (DOTA-(d-Glu)_6_-Ala-Tyr-Gly-Trp-Met-Asp-Phe-NH_2_) [7, 8] were performed.

However, one major drawback of minigastrin analogs with regard to a clinical use was their low in vivo stability. Therefore, Ocak et al*.* performed comparative stability studies in vitro as well as in vivo of various CCK-2R-targeted peptides, including MG11 and CP04. In the course of these experiments, the Asp-Phe, Gly-Trp and Tyr-Gly sequences were identified as the major cleavage sites of minigastrin analogs [8, 9].

In 2018, Klingler et al*.* reported that the substitution of Phe by 1-Nal and Met by (*N*-Me)Nle (Nle: norleucine) in MG11 led to an increased metabolic stability of the resulting compound (DOTA-MGS5) while high CCK-2R affinity was maintained [10]. Furthermore, [^68^ Ga]Ga-DOTA-MGS5 revealed high activity levels in the tumor (23.3 ± 4.7%ID/g), while displaying low activity uptake in the kidneys (5.7 ± 1.4%ID/g) at 1 h p.i. in A431-CCK-2R tumor-bearing nude mice [11, 12]. Further studies on substituting either Ala, Tyr or Gly in DOTA-MGS5 by Pro residues resulted in highly CCK-2R-affine peptides with improved metabolic stability. Especially DOTA-MGS8 (DOTA-d-Glu-Pro-Tyr-Gly-Trp-(*N-*Me)Nle-Asp-1-Nal-NH_2_) led to improved activity levels in the tumor and thus, tumor/background ratios as compared to DOTA-MGS5 [13–15].

Another strategy to stabilize CCK-2R-targeted compounds was attempted by a systematic substitution of peptide bonds by 1,4-disubstituted 1,2,3-triazoles in DOTA-[Nle^15^]MG11 (DOTA-d-Glu-Ala-Tyr-Gly-Trp-Nle-Asp-Phe-NH_2_) and DOTA-PP-F11N (DOTA-(d-Glu)_6_-Ala-Tyr-Gly-Trp-Nle-Asp-Phe-NH_2_). Only a minor impact on CCK-2R affinity was observed for peptides comprising a triazole bond between the d-Glu-Pro, Pro-Tyr, Tyr-Gly and Gly-Trp sequence. However, increased metabolic stability was only observed for compounds that comprise triazole bonds between Trp-Nle, Nle-Asp or Asp-Phe, which led to a loss in CCK-2R affinity, though. Therefore, no noticeable benefit on tumor accumulation was observed [16, 17].

**Fig. 1:**
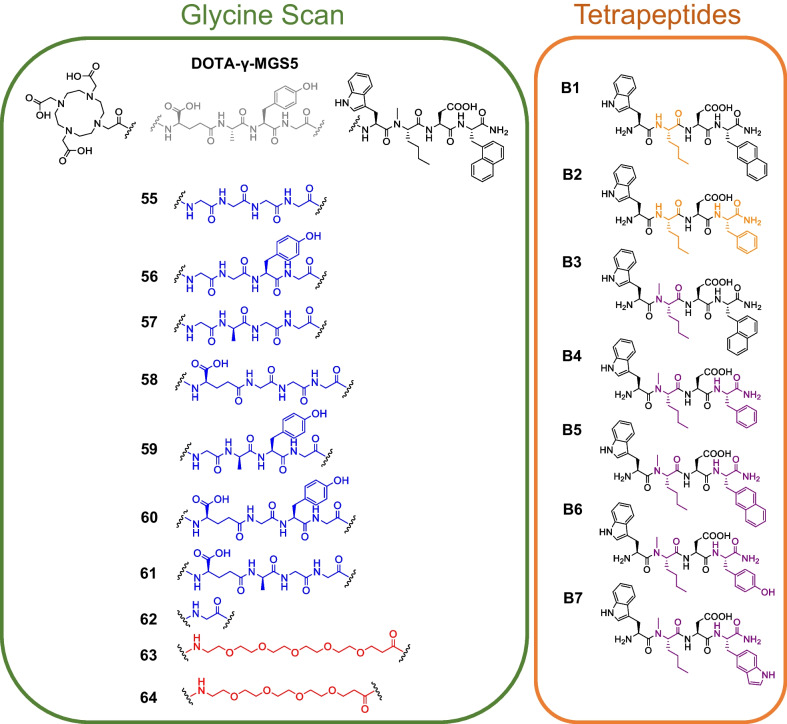
Chemical structures of the glycine scan derivatives (blue), the PEG-linked compounds (red), the tetrapeptides lacking an *N*-methyl group at the norleucine (Nle) moiety (orange) and the tetrapeptides comprising an *N*-methyl group at the Nle moiety (purple)

Considering the above-mentioned studies, a high tolerance toward modifications within minigastrin analogs with regard to CCK-2R affinity was observed, especially when the *N*-terminal sequence (d-Glu-Ala-Tyr-Gly) was addressed [10, 13, 16, 17]. Indeed, substitution of *α*- by *γ*-linked d-glutamate moieties within DOTA-PP-F11N had a beneficial impact on CCK-2R affinity [18]. Due to this high tolerability toward modifications at the *N*-terminus, one aim of this study was to provide a better insight on the structure–activity relationship of minigastrin analogs at this part. Therefore, we carried out a glycine scan to elucidate the influence of the d-γ-Glu-Ala-Tyr-Gly sequence in DOTA-γ-MGS5 (DOTA-D-γ-Glu-Ala-Tyr-Gly-Trp-(*N-*Me)Nle-Asp-1-Nal-NH_2_) on CCK-2R affinity and if a simple polyethylene glycol (PEG) spacer can retain high CCK-2R affinity and concurrently improve metabolic stability (Fig. 1). Furthermore, it was investigated whether the *C*-terminal tetrapeptide is sufficient for high CCK-2R affinity and which impact small modifications within this tetrapeptide unit have on affinity.

## Materials and methods

Characterization of all CCK-2R-targeted compounds is provided in the Additional file 1: (Fig. S1–S17). Electrospray ionization-mass spectra for characterization of the substances were acquired on an expression^L^ CMS mass spectrometer (Advion Ltd., Harlow, UK).

### Chemical synthesis and labeling procedures

Synthesis of the peptide precursors was conducted via solid-phase peptide synthesis (SPPS) using an *H-*Rink amide ChemMatrix® resin (35–100 mesh particle size, 0.4–0.6 mmol/g loading, Merck KGaA, Darmstadt, Germany). After resin cleavage with concomitant cleavage of acid-labile protecting groups, final purification was performed by reversed phase high performance liquid chromatography (RP-HPLC). ^nat^Lu- and ^177^Lu-labeling was conducted according to previously published procedures [19].

### In vitro* experiments*

*IC*_50_ values of all compounds were determined according to a previously published procedure [18]. In brief, competitive binding studies were performed via co-incubation of increasing concentrations of the peptide of interest (10^−10^ to 10^–4^ M, in triplicate) together with the reference compound [^177^Lu]Lu-DOTA-PP-F11N (0.3 pmol/well) on AR42J cells (2 × 10^5^ cells per 1 mL/well) at 37 °C for 3 h.

Lipophilicity (depicted as *n*-octanol-phosphate-buffered saline solution (PBS, *pH* = 7.4) distribution coefficient, log*D*_7.4_) of ^177^Lu-labeled minigastrin analogs was evaluated as previously published [18].

In vitro stability of the ^177^Lu-labeled peptides (1 nmol, ~ 5 MBq) was analyzed via radio-RP-HPLC after incubation at 37 °C for 24 h in human serum of a healthy donor via an established protocol [20].

### Statistics

Acquired data were statistically analyzed by performing a Student’s *t*-test via Excel (Microsoft Corporation, Redmond, WA, USA) and OriginPro software (version 9.7) from OriginLab Corporation (Northampton, MA, USA). Acquired *p* values of < 0.05 were considered statistically significant.

## Results

### Chemical synthesis and radiolabeling

Synthesis via SPPS and subsequent RP-HPLC purification yielded 4–8% off-white solid (chemical purity  >  95%, determined by RP-HPLC at *λ* = 220 nm). Quantitative ^nat^Lu-labeling of the peptides was accomplished by adding a 2.5-fold excess of [^nat^Lu]LuCl_3_ to the DOTA-comprising peptides and heating the solution to 90 °C for 15 min. As unbound Lu^3+^ did not reveal any influence on affinity experiments, no further purification steps were conducted prior to usage [21]. ^177^Lu-labeling proceeded in radiochemical yields and purities of > 95% and molar activities of 10–50 GBq/µmol.

### In vitro evaluation

**Fig. 2:**
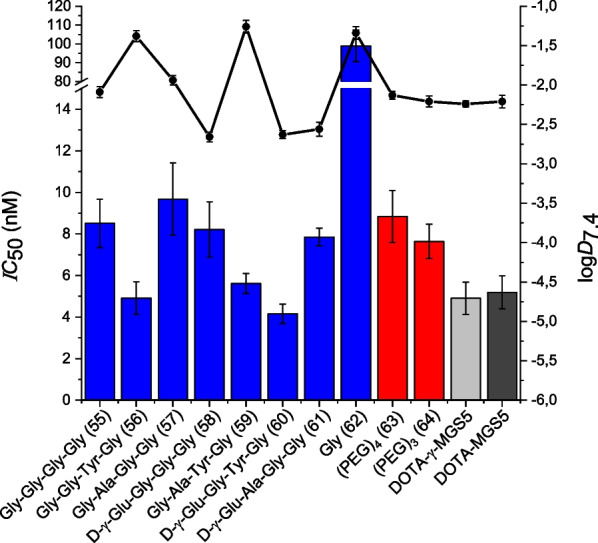
*IC*_50_ and log*D*_7.4_ values of the glycine scan derivatives ([^nat/177^Lu]Lu-DOTA-CCK-55 to − 62, depicted in blue) and peptides containing a PEG spacer unit (depicted in red, [^nat/177^Lu]Lu-DOTA-CCK-63 and -64), as well as the reference ligands [^nat/177^Lu]Lu-DOTA-MGS5 (depicted in light gray) and [^nat/177^Lu]Lu-DOTA-γ-MGS5 (depicted in dark gray)

Affinity and lipophilicity data of the glycine scan derivatives are summarized in Fig. 2.

In general, [^nat^Lu]Lu-DOTA-CCK-55 to -61 revealed *IC*_50_ values (4.2–9.7 nM, Additional file 1: Table S1) in a low nanomolar range, comparable to those of the reference compounds [^nat^Lu]Lu-DOTA-MGS5 (5.2 ± 0.8 nM) and [^nat^Lu]Lu-DOTA-γ-MGS5 (4.9 ± 0.8 nM), while [^nat^Lu]Lu-DOTA-CCK-62, a compound completely lacking the amino acid sequence D-γ-Glu-Ala-Tyr, exhibited a significant loss in CCK-2R affinity (*IC*_50_ = 98.9 ± 8.4 nM, *p* < 0.0001). Substitution of the amino acid sequence Dγ-Glu-Ala-Tyr-Gly by a (PEG)_4_ or (PEG)_3_ linker ([^nat^Lu]Lu-DOTA-CCK-63 and -64, respectively) had only a minor impact on CCK-2R affinity (*IC*_50_: 8.8 ± 1.3 and 7.6 ± 0.9 nM).

Log*D*_7.4_ values of [^177^Lu]Lu-DOTA-CCK-55, -58, -60, -61, -63, and -64 were observed to be in a range of ‒2.7 to ‒2.1, similar to the references [^177^Lu]Lu-DOTA-MGS5 (log*D*_7.4_ = − 2.21 ± 0.08) and [^177^Lu]Lu-DOTA-γ-MGS5 (log*D*_7.4_ = − 2.24 ± 0.04). In contrast, [^177^Lu]Lu-DOTA-CCK-56, − 57, − 59 and − 62 were found to be more lipophilic (log*D*_7.4_ = − 1.9 to − 1.2).

**Fig. 3:**
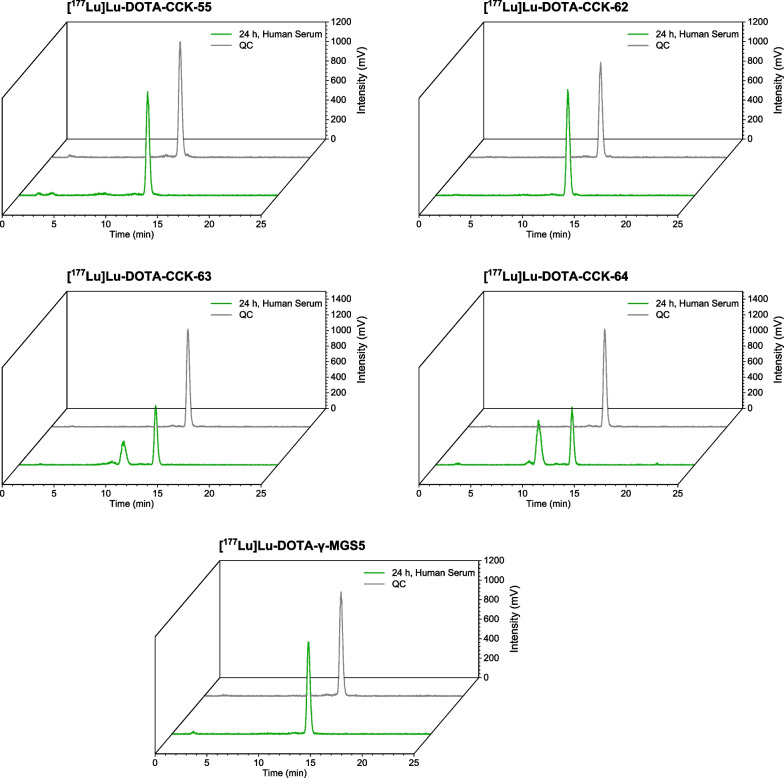
In vitro stability studies in human serum (24 h, 37 °C) as analyzed by analytical RP-HPLC (10 → 30% MeCN in H_2_O + 0.1% TFA in 5 min, 30 → 60% MeCN in H_2_O + 0.1% TFA in 5 min)

Stability studies revealed high in vitro stability in human serum (incubation for 24 h at 37 °C, Fig. 3, Additional file 1: Table S2) for both [^177^Lu]Lu-DOTA-γ-MGS5 (96.8 ± 2.8%) as well as [^177^Lu]Lu-DOTA-CCK-55 (87.5 ± 1.9%) and − 62 (97.9 ± 1.8%), which comprised a (Gly)_4_ and Gly, respectively, instead of a d-γ-Glu-Ala-Tyr-Gly linker (as in [^177^Lu]Lu-DOTA-γ-MGS5). In contrast, [^177^Lu]Lu-DOTA-CCK-63 (57.4 ± 3.7%) and − 64 (43.8 ± 2.3%) that contain a (PEG)_4_ and (PEG)_3_ moiety, respectively, instead of a d-γ-Glu-Ala-Tyr-Gly linker, displayed distinctly lower in vitro stability than the other compounds.

**Fig. 4:**
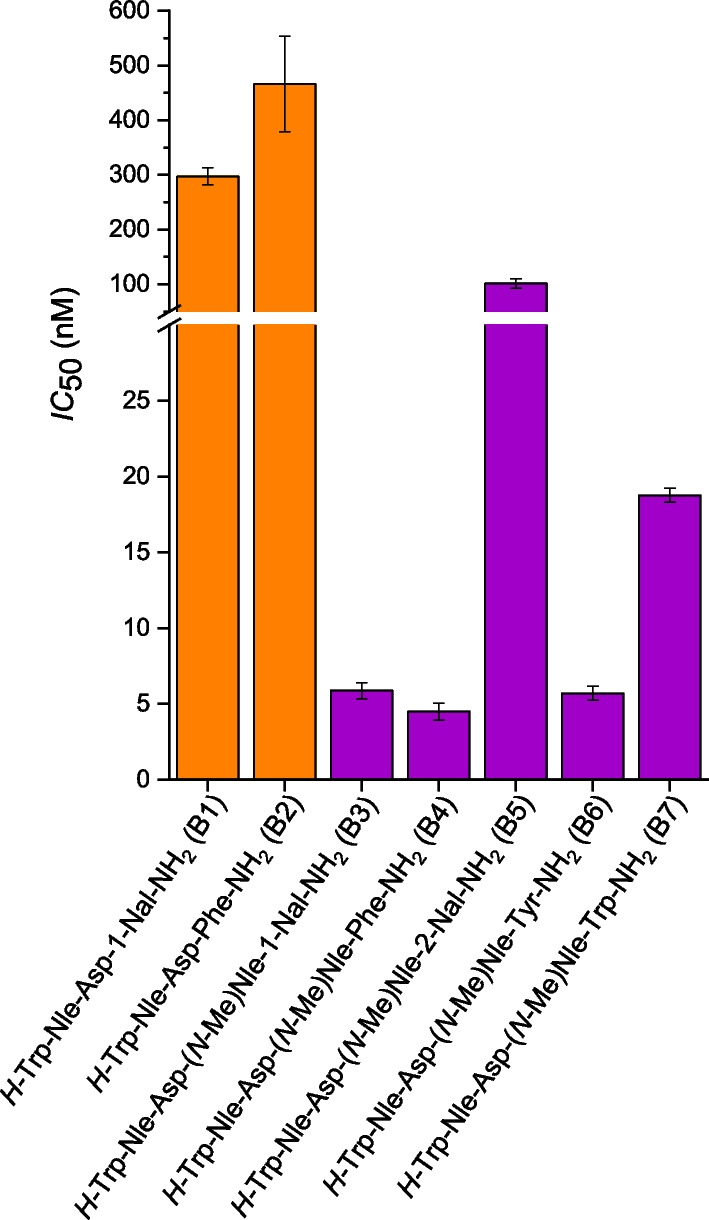
*IC*_50_ values of various tetrapeptide CCK-2R binding sequences

In order to evaluate whether a *C*-terminal tetrapeptide is sufficient for high CCK-2R affinity, we determined the *IC*_50_ values of various tetrapeptides (Fig. 4, Additional file 1: Table S1).

B3, B4 and B6, which contain an *N*-methyl group at the L-Nle moiety revealed *IC*_50_ values in a low nanomolar range (4.5–5.9 nM), whereas B1 and B2, which did not comprise an *N*-methylated Nle residue, exhibited a significant loss in CCK-2R affinity (*p* < 0.0001). However, B5 and B7, comprising either an L-2-naphtylalanine or L-tryptophan moiety at the *N*-terminus of the tetrapeptide sequence demonstrated 3.5- to 19-fold elevated *IC*_50_ values compared to B3, B4 and B6, despite carrying an *N*-methylated L-Nle.

## Discussion

Over the years, the effect of various modifications in minigastrin analogs on CCK-2R affinity and in vivo stability was investigated. Especially the *N*-terminal amino acids d-Glu, Ala, Tyr and Gly were substituted by different building blocks, such as proline moieties (Klingler et al*.*) or triazole bonds (Grob et al*.*), which did not result in a major loss in CCK-2R affinity [13, 16, 17]. As we wanted to examine whether the presence of all of these four amino acids (d-Glu, Ala, Tyr and Gly) is crucial for high CCK-2R affinity, one aim of this study was to systematically substitute said amino acids by glycine to elucidate the structure–activity relationship of these amino acids within minigastrin analogs. As recently published data by our group suggested a beneficial impact of γ- instead of α-linked D-glutamate moieties on CCK-2R affinity [18], we carried out our studies on the peptide DOTA-γ-MGS5 (DOTA- d-γ-Glu-Ala-Tyr-Gly-Trp-(*N*-Me)Nle-Asp-1-Nal-NH_2_). Furthermore, we wanted to investigate whether the *C*-terminal tetrapeptide (*H*-Trp-(*N*-Me)Nle-Asp-1-Nal-NH_2_) might be sufficient for high CCK-2R affinity.

The glycine scan revealed that D-γ-Glu, Ala and Tyr each can be substituted by a Gly, without causing a major loss in CCK-2R affinity (*IC*_50_ = 4.2–9.7 nM), in comparison to the references [^nat^Lu]Lu-DOTA-MGS5 (*IC*_50_ = 5.2 ± 0.8 nM) and [^nat^Lu]Lu-DOTA-γ-MGS5 (*IC*_50_ = 4.9 ± 0.8 nM). Therefore, it can be concluded that these three amino acids are not necessary for high CCK-2R affinity, as each could be easily replaced by a glycine residue. Similar observations were made by Silvente-Poirot et al*.* when successively substituting each amino acid in CCK2–9 (*H*-Asp-Tyr-Met-Gly-Trp-Met-Asp-Phe-NH_2_) by an alanine residue, which exhibited only slight loss of CCK-2R affinity for the substitution of the *N*-terminal amino acids, while substitution of the four *C*-terminal amino acids resulted in low CCK-2R affinity [22]. These data accompany previously reported data on modifications at the *N*-terminal part of minigastrin analogs, as it could be demonstrated that the tolerability toward substitutions is high [14, 17, 18]. However, it could be shown that the presence of these amino acids is required with regard to the distance of the DOTA chelator to the pharmacophore, as [^nat^Lu]Lu-DOTA-CCK-62 (DOTA-Gly-Trp-(*N-*Me)Nle-Asp-1-Nal-NH_2,_ *IC*_50_ = 98.9 ± 8.4 nM) revealed a significant loss in CCK-2R affinity (*p* < 0.0001). Hence, a spacer unit between the binding motif and the chelator moiety is necessary, in order to retain high CCK-2R binding. Nevertheless, it is legitimate to question whether the pharmacophore consists of only four instead of seven amino acids, as the three *N*-terminal amino acids could be replaced by glycine residues, which are usually not linked to a pharmacological effect. This is supported by Silvente-Poirot et al*.* who showed high CCK-2R affinity for the *H*-Trp-Met-Asp-Phe-NH_2_ fragment [22].

In order to further examine this assumption, we substituted D-γ-Glu-Ala-Tyr-Gly in DOTA-γ-MGS5 by a (PEG)_4_ as well as a (PEG)_3_ spacer, which resulted in slightly less (compared to the references) yet still highly CCK-2R-affine minigastrin analogs ([^nat^Lu]Lu-DOTA-CCK-63: *IC*_50_ = 8.8 ± 1.3 nM, [^nat^Lu]Lu-DOTA-CCK-64: *IC*_50_ = 7.6 ± 0.9 nM). The loss of affinity can be attributed to the lack of negative charges within the linker section, as it was shown that the CCK-2R comprises a high number of positively charged residues in the region that interacts with the linker section of its ligands, which is why negatively charged moieties at the *N*-terminus usually lead to increased CCK-2R affinity [23]. Supported by our results, we strongly suggest that the pharmacophore of minigastrin analogs indeed consists of only four amino acids, while the remaining *N*-terminal amino acids such as d-γ-Glu or D-Glu, Ala, Tyr and Gly can be mainly considered a spacer for the (DOTA) chelator.

With the replacement of four *N*-terminal amino acids (of which three are L-amino acids) by an unnatural PEG chain, we aimed to improve metabolic stability of minigastrin analogs, as this should hamper their enzymatic cleavage at two major cleavage sites reported in the literature (Tyr-Gly and Gly-Trp) [8]. However, while high in vitro stability was observed in human serum for the reference, [^177^Lu]Lu-DOTA-γ-MGS5, as well as [^177^Lu]Lu-DOTA-CCK-55 (-(Gly)_3_-) and -62 (-Gly-), a distinctly lower stability was determined for [^177^Lu]Lu-DOTA-CCK-63 (-(PEG)_4_-) and -64 (-(PEG)_3_-), which was surprising, given the robustness of a PEG chain and its usually positive impact on stability [24–27] and the susceptibility of L-amino acids toward in vitro and in vivo degradation. An experimental error can be excluded, as all compounds were incubated (in separate vials) at the same time using the same batch of human serum. The respective metabolites observed for the two PEG-comprising compounds could not be identified, which is why further studies have to be carried out to elucidate the reason for this unexpected observation and to find a strategy to increase metabolic stability of such PEG-containing minigastrin derivatives.

Nevertheless, to further prove that a tetrapeptide motif is sufficient for high CCK-2R affinity, we determined the *IC*_50_ values of various tetrapeptides based on the *C*-terminus of DOTA-(γ-)MGS5, which confirmed our assumption, as the peptides B3 (*H*-Trp-(*N-*Me)Nle-Asp-1-Nal-NH_2_), B4 (*H-*Trp-(*N-*Me)Nle-Asp-Phe-NH_2_) and B6 (*H-*Trp-(*N-*Me)Nle-Asp-Tyr-NH_2_) exhibited CCK-2R affinities in the low nanomolar range. Moreover, it could be demonstrated that the *C*-terminal position tolerates various aromatic residues, such as phenylalanine, L-1-naphtylalanine and tyrosine but not L-2-naphtylalanine or tryptophan. This observation confirmed that there is some tolerability at the *C*-terminus despite options for modifications within the pharmacophore are usually scarce, as even small changes can cause a distinct loss of affinity. Interestingly, when we extended our tetrapeptide analysis to DOTA-PP-F11N, a minigastrin analog currently tested in clinical trials [28], we found that the respective tetrapeptide (*H*-Trp-Nle-Asp-Phe-NH_2_) revealed a noticeably decreased CCK-2R affinity, while this sequence in combination with a linker sequence, such as (d-Glu)_6_-Ala-Tyr-Gly showed high affinity [18, 29]. This was surprising, as Silvente-Poirot et al*.* reported high CCK-2R affinity for the tetrapeptide *H*-Trp-Met-Asp-Phe-NH_2_ [22].

As the tetrapeptide B1 (*H*-Trp-Nle-Asp-1-Nal-NH_2_) also displayed low CCK-2R affinity, we suspected that *N-*methylation at the Nle moiety is crucial for high CCK-2R affinity of Nle-comprising tetrapeptides. In fact, B1 containing a Nle moiety displayed a 50-fold higher *IC*_50_ value than B3 comprising an *N-*methylated Nle moiety, while the remaining sequence was identical. We thus conclude that a tetrapeptide motif at the *C*-terminus of minigastrin derivatives can be sufficient when containing a Nle moiety but seems to require an *N*-methylated peptide backbone at the Nle site, which is the most substantial finding of this work. We are aware that Silvente-Poirot et al*.* showed high CCK-2R affinity for the tetrapeptide *H*-Trp-Met-Asp-Phe-NH_2_, which does not contain any *N*-methylation [22]. However, as Met is usually replaced by a Nle moiety due to its susceptibility toward in vivo oxidation and thus in vivo degradation, we believe that our results could affect the future design of CCK-2R-targeted compounds.

Not surprisingly, log*D*_7.4_ values confirmed the positive impact of a negatively charged D-γ-Glu moiety on lipophilicity. [^177^Lu]Lu-DOTA-CCK-63 and -64, comprising either a (PEG)_4_ or (PEG)_3_ spacer instead of d-γ-Glu-Ala-Tyr-Gly, still revealed comparable log*D*_7.4_ values to those of the references [^177^Lu]Lu-DOTA-MGS5 and [^177^Lu]Lu-DOTA-γ-MGS5 (− 2.13 ± 0.05 and − 2.21 ± 0.07 *versus* − 2.21 ± 0.08 and − 2.24 ± 0.04, respectively), which suggests a positive impact of the PEG spacer on lipophilicity.

## Conclusion

We could confirm that the amino acid sequence d-γ-Glu-Ala-Tyr-Gly of minigastrin analogs is not required for high CCK-2R affinity but can be rather considered a spacer. Hence, substitution by PEG spacers simplified the peptide structure while maintaining high CCK-2R affinity and sufficient lipophilicity. In addition, we could confirm that only the tetrapeptide amino acid sequence *H*-Trp-(*N-*Me)Nle-Asp-1-Nal-NH_2_ in DOTA-MGS5 is required for high CCK-2R affinity, yet the presence of a tiny *N*-methyl group at the peptide backbone of the Nle moiety is crucial, which could affect future design of CCK-2R-targeted compounds. However, our initial rationale that substitution of the *N*-terminal L-amino acids Ala-Tyr-Gly by unnatural PEG chains would increase metabolic stability could not be confirmed. This unexpected finding has to be further investigated in future studies, particularly whether the introduction of additional moieties in these PEG-containing minigastrin analogs could improve metabolic stability while maintaining the beneficial aspects of PEGylation observed in this study.

## Supplementary Information

**Additional file 1**. Characterization of all CCK-2R-targeted compounds (Figure S1-S17) evaluated in this work, as well as additional information on CCK-2R affinity, lipophilicity (Table S1) and stability in human serum (Table S2).

## Data Availability

Data are contained within the article and Additional file 1.

## Abbreviations

CCK-2R: Cholecystokinin-2 receptor
CP04: DOTA-(d-Glu)6-Ala-Tyr-Gly-Trp-Met-Asp-Phe-NH_2_
DOTA: 1,4,7,10-Tetraazacyclododecan-1,4,7,10-tetracetic acid
DOTA-MGS5: DOTA-d-Glu-Ala-Tyr-Gly-Trp-(N-Me)Nle-Asp-1-Nal-NH_2_
DOTA-MGS8: DOTA- d-Glu-Pro-Tyr-Gly-Trp-(N-Me)Nle-Asp-1-Nal-NH_2_
DOTA-[Nle^15^]MG11: DOTA- d-Glu-Ala-Tyr-Gly-Trp-Nle-Asp-Phe-NH_2_
DOTA-PP-F11N: DOTA-(d-Glu)_6_-Ala-Tyr-Gly-Trp-Nle-Asp-Phe-NH_2_
DTPA: Diethylenetriaminepentaacetic acid
DTPA-MG0: DTPA- d-Glu-(GIu)_5_-Ala-Tyr-Gly-Trp-Met-Asp-Phe-NH_2_
ESI–MS: Electro-spray ionization mass spectrometry
Fmoc: 9-Fluorenylmethoxycarbonyl
MG11: H-d-Glu-Ala-Tyr-Gly-Trp-Met-Asp-Phe-NH_2_
MTC: Medullary thyroid carcinoma
PEG: Polyethylene glycol
radio-TLC: Radio-thin layer chromatography
RCP: Radiochemical purity
RCY: Radiochemical yield
RP-HPLC: Reversed-phase high performance liquid chromatography
SPPS: Solid-phase peptide synthesis
