# Supplementary material for: Investigation of the structure-activity relationship at the N-terminal part of minigastrin analogs
Source: EJNMMI Res. 2023 Jul 8;13:65. doi: 10.1186/s13550-023-01016-y (PMC10329608; doi:10.1186/s13550-023-01016-y)
Supplement: Supplementary file 1 — Additional file 1. Characterization of all CCK-2R-targeted compounds (Figure S1-S17) evaluated in this work, as well as additional information on CCK-2R affinity, lipophilicity (Table S1) and stability in human serum (Table S2). [file 13550_2023_1016_MOESM1_ESM.docx]

**Investigation of the Structure-Activity Relationship at the *N*-terminal part of Minigastrin Analogs**

**- Supplementary Materials -**

Nadine Holzleitner^1,#,^*, Thomas Günther^1, #,^*, Amira Daoud-Gadieh^1^, Constantin Lapa^2^ and Hans-Jürgen Wester^1^

^1^ Technical University of Munich, Department of Chemistry, 85748 Garching, Germany

^2^ University Hospital Augsburg, Nuclear Medicine, 86156 Augsburg, Germany

^#^ both authors contributed equally

* Corresponding author: Thomas Günther, thomas.guenther@tum.de, Technical University of Munich, Department of Chemistry, Garching, Germany

Nadine Holzleitner, nadine.holzleitner@tum.de, Technical University of Munich, Department of Chemistry, Garching, Germany

**Corresponding authors:**

Thomas Günther and Nadine Holzleitner

Phone: +49.89.289.12203

Technical University of Munich,

Chair of Pharmaceutical Radiochemistry,

Walther-Meissner-Str. 3

85748 Garching

GERMANY

Fax: +49.89.289.12204

E-Mail: [thomas.guenther@tum.de](mailto:thomas.guenther@tum.de) and [nadine.holzleitner@tum.de](mailto:nadine.holzleitner@tum.de)

ORCID: <https://orcid.org/0000-0002-7412-0297> (TG) and <https://orcid.org/0000-0001-8258-3526> (NH)

**Analytical data of ^nat/177^Lu-labeled minigastrin analogs**

(a)

(b)

(c)


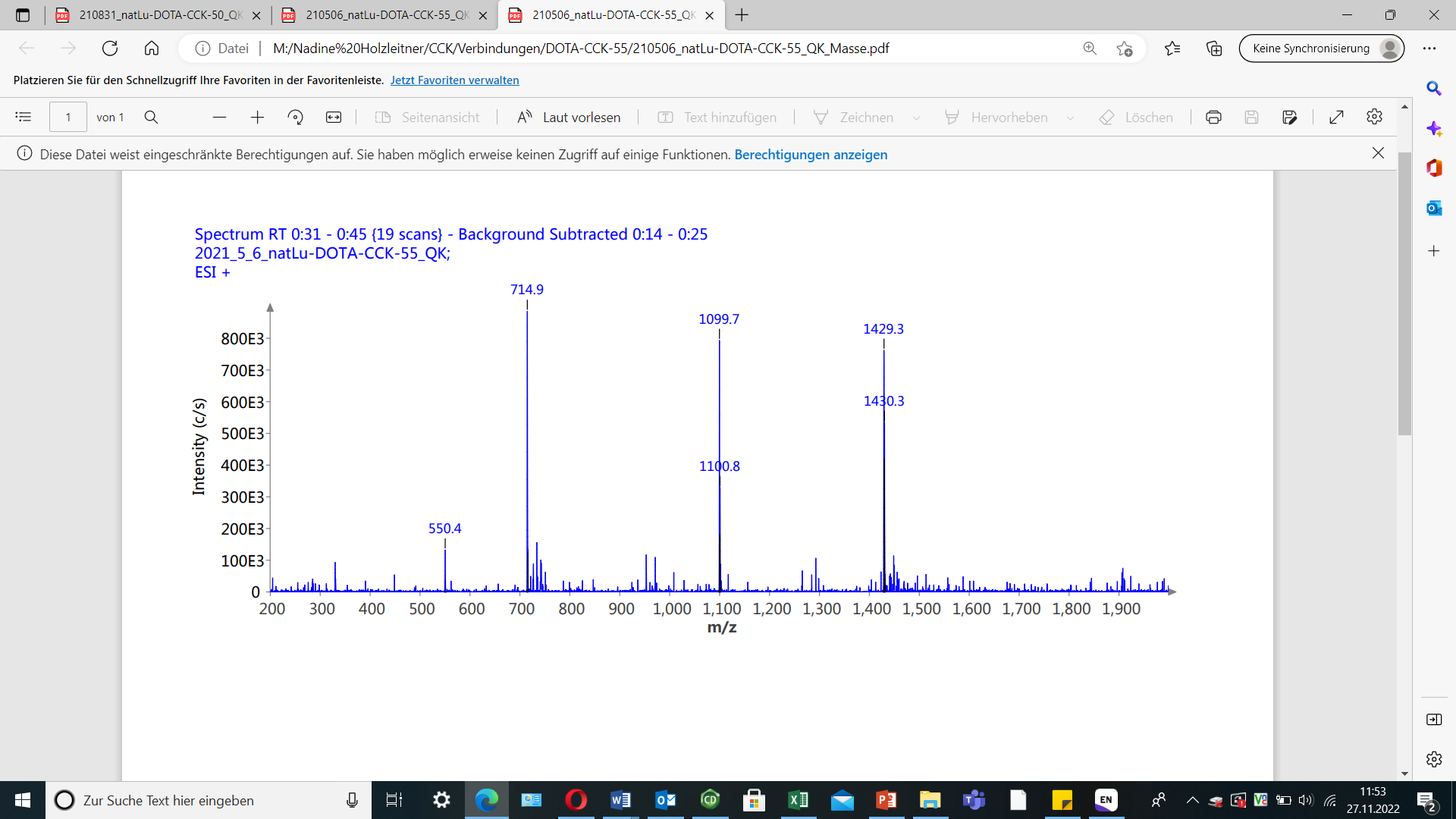


**Figure S1.** Confirmation of peptide identity and integrity for (**a**) [^nat^Lu]Lu-DOTA-CCK-55 and (**b**) [^177^Lu]Lu-DOTA-CCK-55 as analyzed by analytical (radio-)RP-HPLC (MultoKrom 100-5 C18, 5 μm, 125 × 4.6 mm, CS Chromatographie GmbH, Langerwehe, Germany; 10→70% MeCN in H_2_O + 0.1% TFA in 15 min). (**c**) Mass spectrum of [^nat^Lu]Lu-DOTA-CCK-55.

***[^nat^Lu]Lu-DOTA-CCK-55***: RP-HPLC (10→70% MeCN in H_2_O with 0.1% TFA, 15 min, λ = 220 nm): *t*_R_ = 10.9 min, K’ = 5.46; MS (ESI, positive): m/z calculated for C_59_H_77_LuN_14_O_17_: 1429.3, found: m/z = 1429.3 [M+H]^+^, 714.9 [M+2H]^2+^.

(a)

(b)

(c)


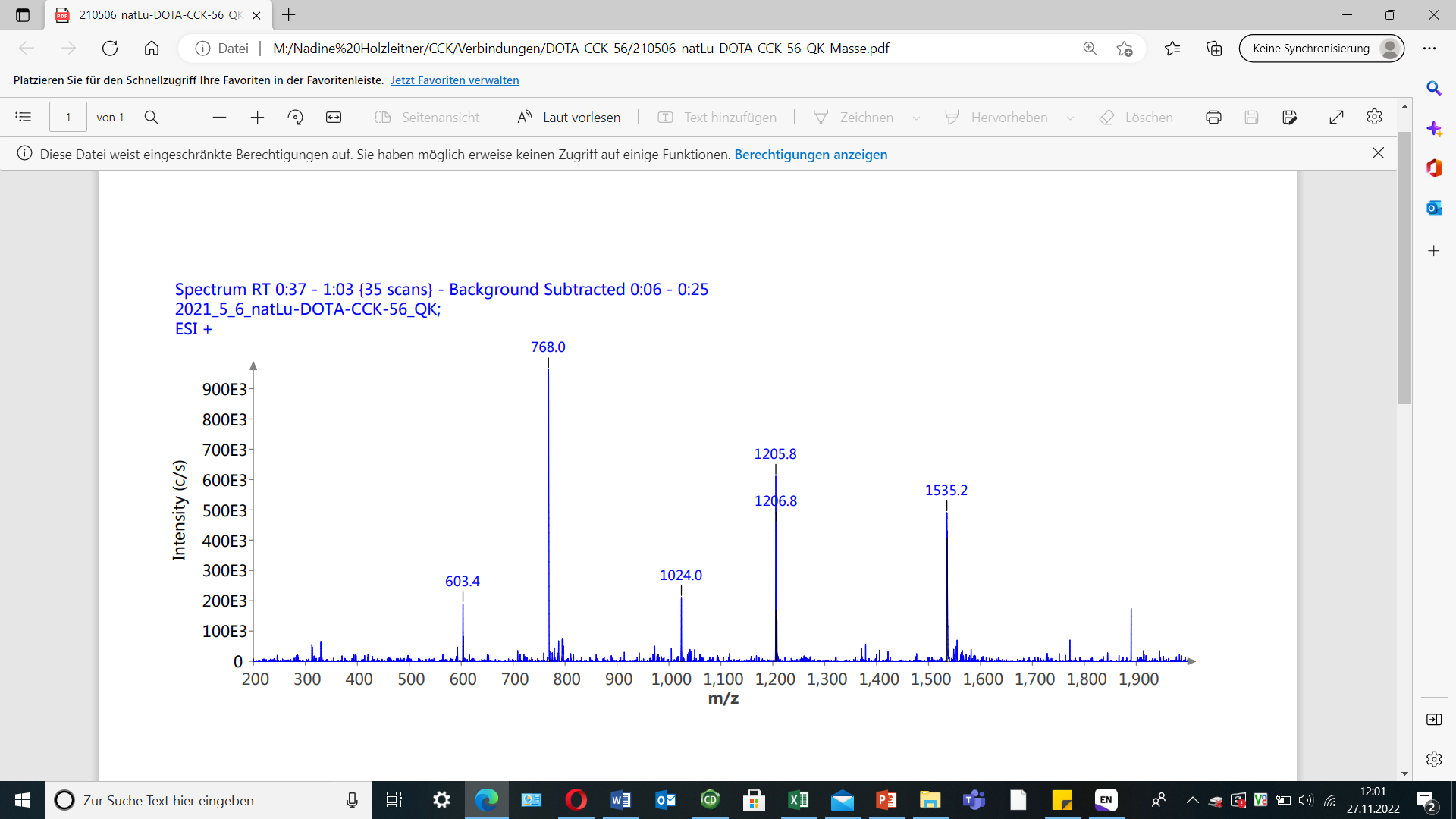


**Figure S2.** Confirmation of peptide identity and integrity for (**a**) [^nat^Lu]Lu-DOTA-CCK-56 and (**b**) [^177^Lu]Lu-DOTA-CCK-56 as analyzed by analytical (radio-)RP-HPLC (MultoKrom 100-5 C18, 5 μm, 125 × 4.6 mm, CS Chromatographie GmbH, Langerwehe, Germany; 10→70% MeCN in H_2_O + 0.1% TFA in 15 min). (**c**) Mass spectrum of [^nat^Lu]Lu-DOTA-CCK-56.

***[^nat^Lu]Lu-DOTA-CCK-56***: RP-HPLC (10→70% MeCN in H_2_O with 0.1% TFA, 15 min, λ = 220 nm): *t*_R_ = 11.1 min, K’ = 5.58; MS (ESI, positive): m/z calculated for C_56_H_83_LuN_14_O_18_: 1535.4, found: m/z = 1535.2 [M+H]^+^, 768.0 [M+2H]^2+^.

(a)

(b)

(c)


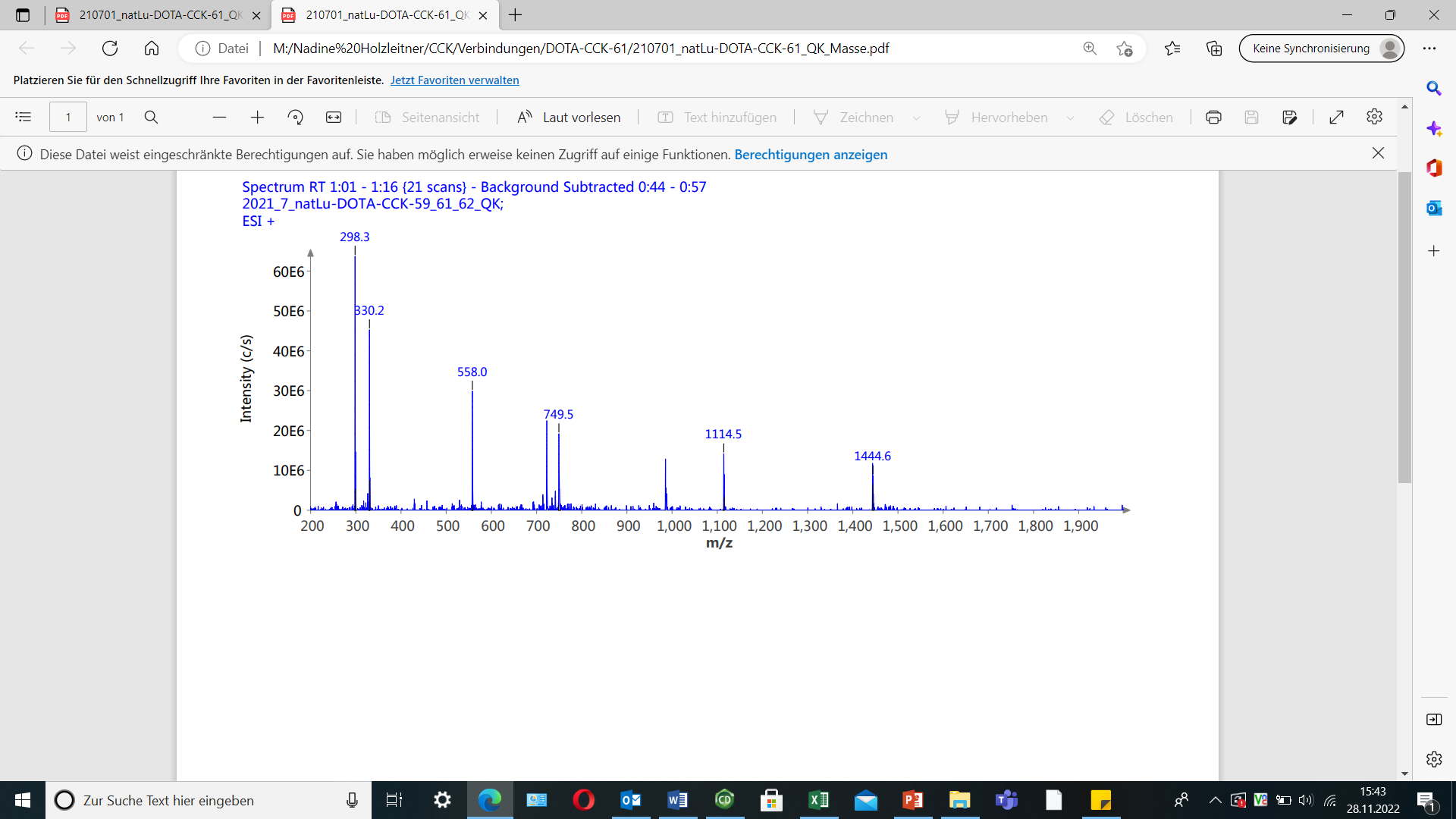


**Figure S3.** Confirmation of peptide identity and integrity for (**a**) [^nat^Lu]Lu-DOTA-CCK-57 and (**b**) [^177^Lu]Lu-DOTA-CCK-57 as analyzed by analytical (radio-)RP-HPLC (MultoKrom 100-5 C18, 5 μm, 125 × 4.6 mm, CS Chromatographie GmbH, Langerwehe, Germany; 10→70% MeCN in H_2_O + 0.1% TFA in 15 min). (**c**) Mass spectrum of [^nat^Lu]Lu-DOTA-CCK-57.

***[^nat^Lu]Lu-DOTA-CCK-57***: RP-HPLC (10→70% MeCN in H_2_O with 0.1% TFA, 15 min, λ = 220 nm): *t*_R_ = 11.0 min, K’ = 5.52; MS (ESI, positive): m/z calculated for C_60_H_79_LuN_14_O_17_: 1443.3, found: m/z = 1444.6 [M+H]^+^.

(a)

(b)

(c)


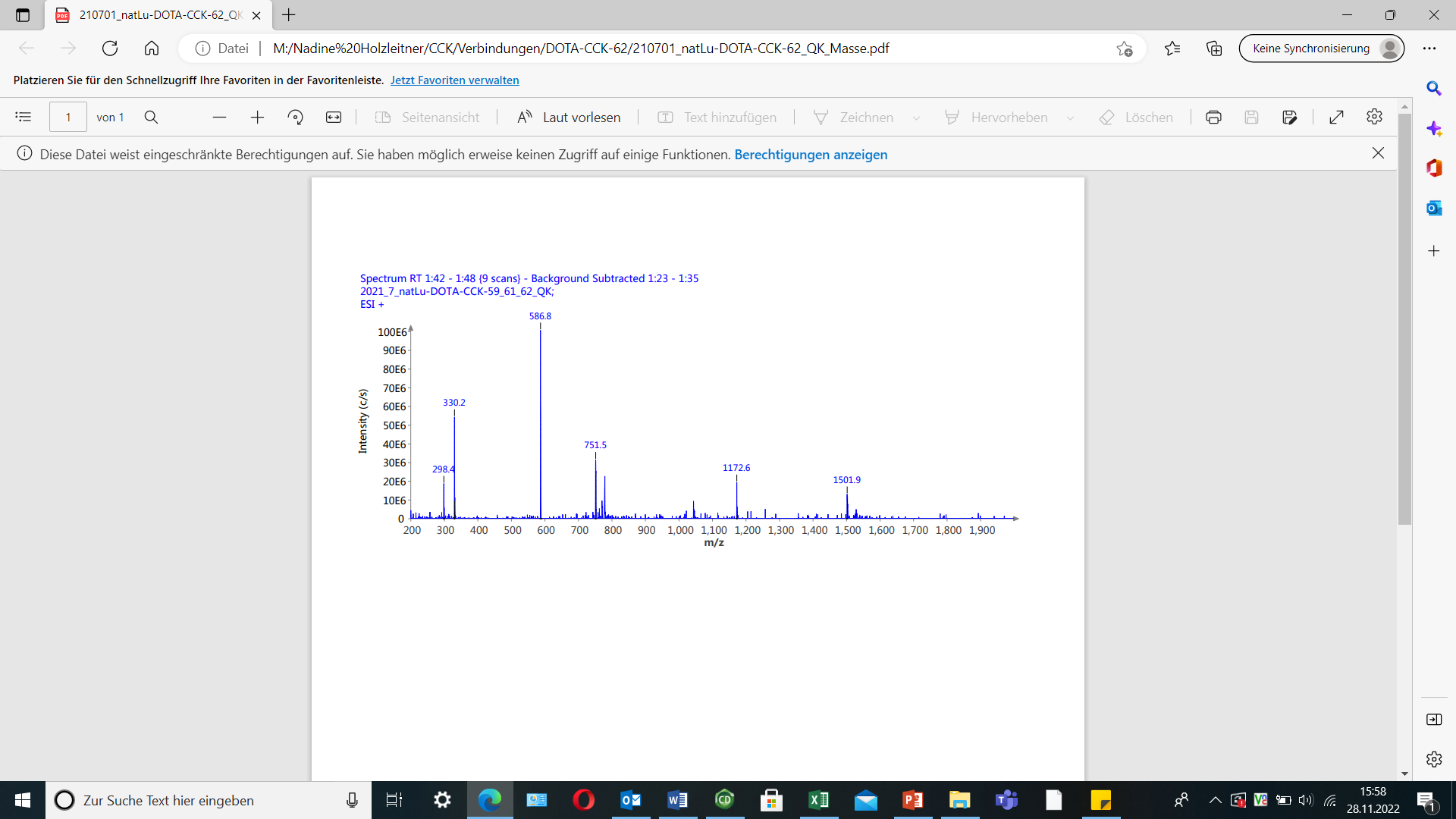


**Figure S4.** Confirmation of peptide identity and integrity for (**a**) [^nat^Lu]Lu-DOTA-CCK-58 and (**b**) [^177^Lu]Lu-DOTA-CCK-58 as analyzed by analytical (radio-)RP-HPLC (MultoKrom 100-5 C18, 5 μm, 125 × 4.6 mm, CS Chromatographie GmbH, Langerwehe, Germany; 10→70% MeCN in H_2_O + 0.1% TFA in 15 min). (**c**) Mass spectrum of [^nat^Lu]Lu-DOTA-CCK-58.

***[^nat^Lu]Lu-DOTA-CCK-58***: RP-HPLC (10→70% MeCN in H_2_O with 0.1% TFA, 15 min, λ = 220 nm): *t*_R_ = 10.8 min, K’ = 5.40; MS (ESI, positive): m/z calculated for C_62_H_81_LuN_14_O_19_: 1501.4, found: m/z = 1501.9 [M+H]^+^, 751.5 [M+2H]^2+^.

(a)

(b)

(c)


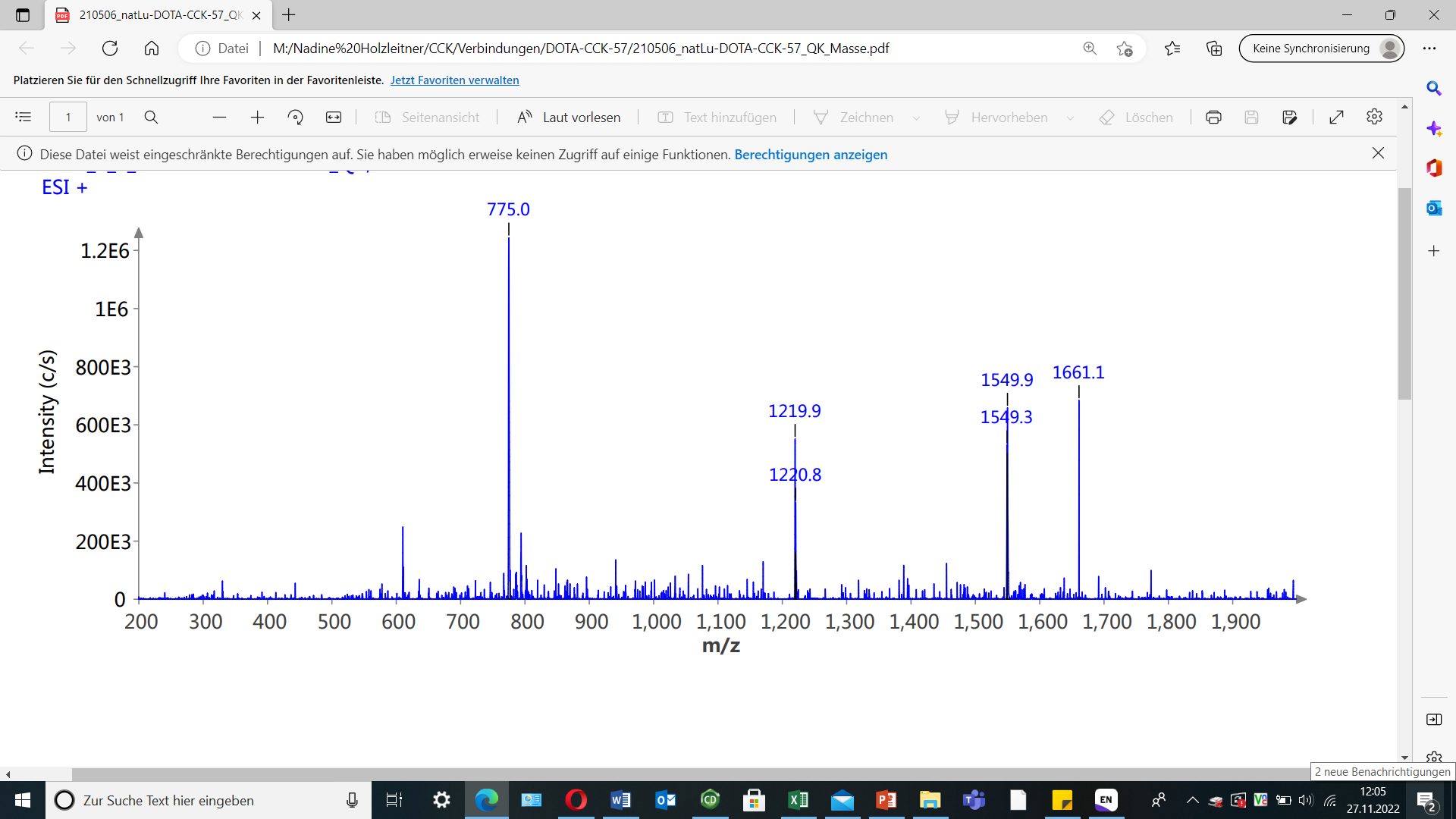


**Figure S5.** Confirmation of peptide identity and integrity for (**a**) [^nat^Lu]Lu-DOTA-CCK-59 and (**b**) [^177^Lu]Lu-DOTA-CCK-59 as analyzed by analytical (radio-)RP-HPLC (MultoKrom 100-5 C18, 5 μm, 125 × 4.6 mm, CS Chromatographie GmbH, Langerwehe, Germany; 10→70% MeCN in H_2_O + 0.1% TFA in 15 min). (**c**) Mass spectrum of [^nat^Lu]Lu-DOTA-CCK-59.

***[^nat^Lu]Lu-DOTA-CCK-59***: RP-HPLC (10→70% MeCN in H_2_O with 0.1% TFA, 15 min, λ = 220 nm): *t*_R_ = 11.2 min, K’ = 5.64; MS (ESI, positive): m/z calculated for C_67_H_85_LuN_14_O_18_: 1549.5, found: m/z = 1549.9 [M+H]^+^, 775.0 [M+2H]^2+^.

(a)

(b)

(c)


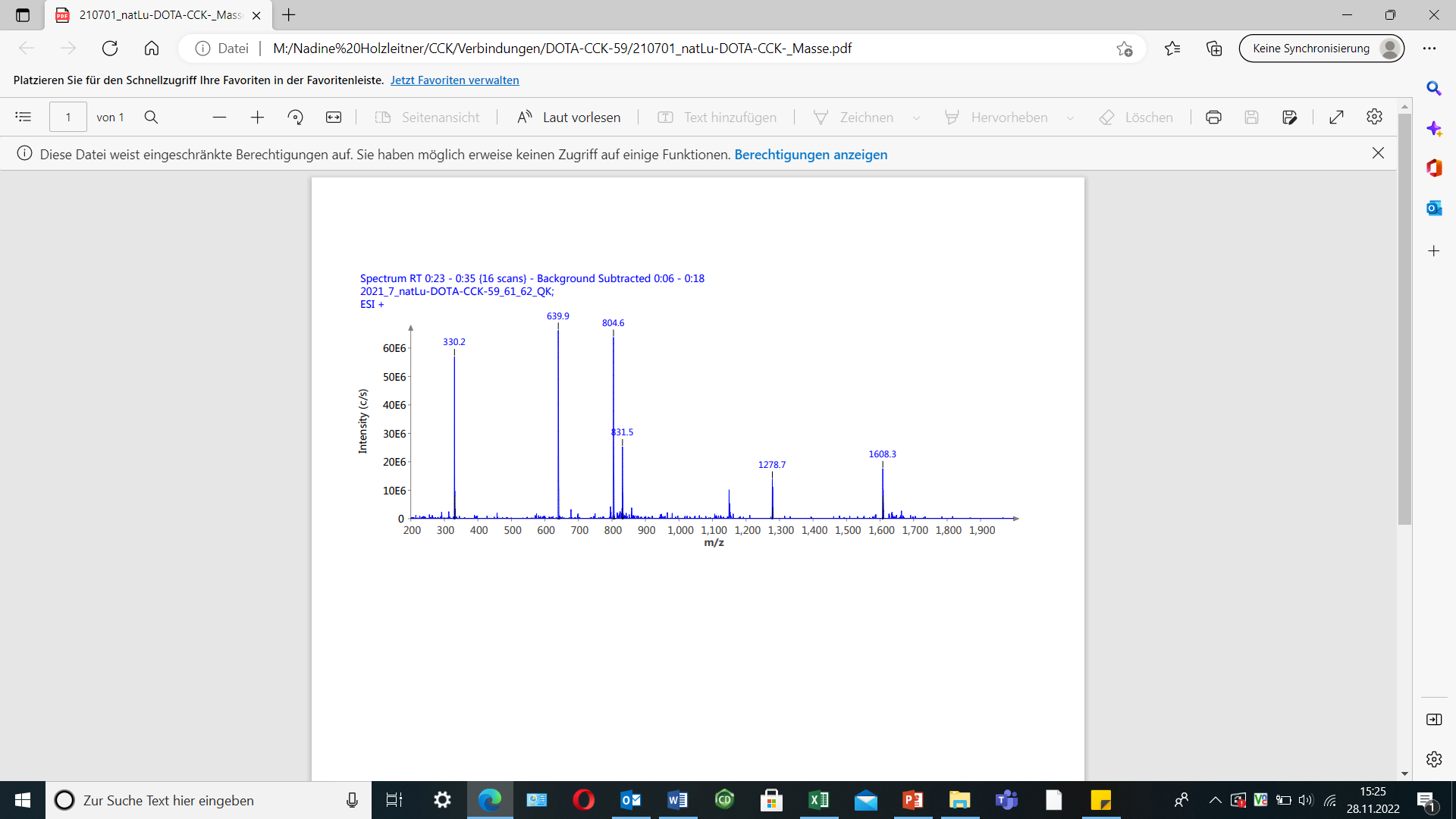


**Figure S6.** Confirmation of peptide identity and integrity for (**a**) [^nat^Lu]Lu-DOTA-CCK-60 and (**b**) [^177^Lu]Lu-DOTA-CCK-60 as analyzed by analytical (radio-)RP-HPLC (MultoKrom 100-5 C18, 5 μm, 125 × 4.6 mm, CS Chromatographie GmbH, Langerwehe, Germany; 10→70% MeCN in H_2_O + 0.1% TFA in 15 min). (**c**) Mass spectrum of [^nat^Lu]Lu-DOTA-CCK-60.

***[^nat^Lu]Lu-DOTA-CCK-60***: RP-HPLC (10→70% MeCN in H_2_O with 0.1% TFA, 15 min, λ = 220 nm): *t*_R_ = 11.1 min, K’ = 5.58; MS (ESI, positive): m/z calculated for C_69_H_87_LuN_14_O_20_: 1607.5, found: m/z = 1608.3 [M+H]^+^, 804.6 [M+2H]^2+^.

(a)

(b)

(c)


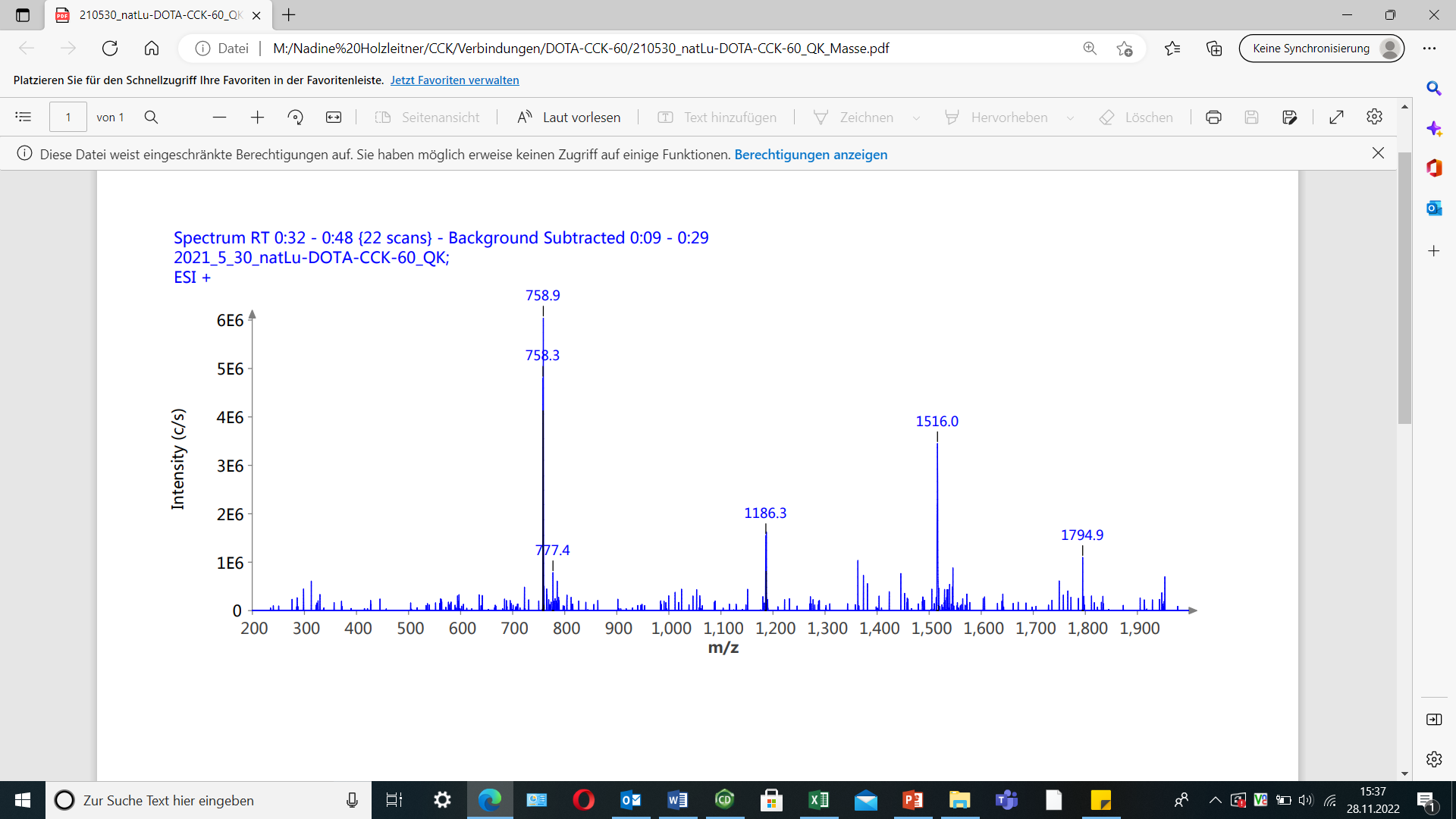


**Figure S7.** Confirmation of peptide identity and integrity for (**a**) [^nat^Lu]Lu-DOTA-CCK-61 and (**b**) [^177^Lu]Lu-DOTA-CCK-61 as analyzed by analytical (radio-)RP-HPLC (MultoKrom 100-5 C18, 5 μm, 125 × 4.6 mm, CS Chromatographie GmbH, Langerwehe, Germany; 10→70% MeCN in H_2_O + 0.1% TFA in 15 min). (**c**) Mass spectrum of [^nat^Lu]Lu-DOTA-CCK-61.

***[^nat^Lu]Lu-DOTA-CCK-61***: RP-HPLC (10→70% MeCN in H_2_O with 0.1% TFA, 15 min, λ = 220 nm): *t*_R_ = 10.8 min, K’ = 5.40; MS (ESI, positive): m/z calculated for C_63_H_83_LuN_14_O_19_: 1515.4, found: m/z = 1516.0 [M+H]^+^, 758.9 [M+2H]^2+^.

(a)

(b)

(c)


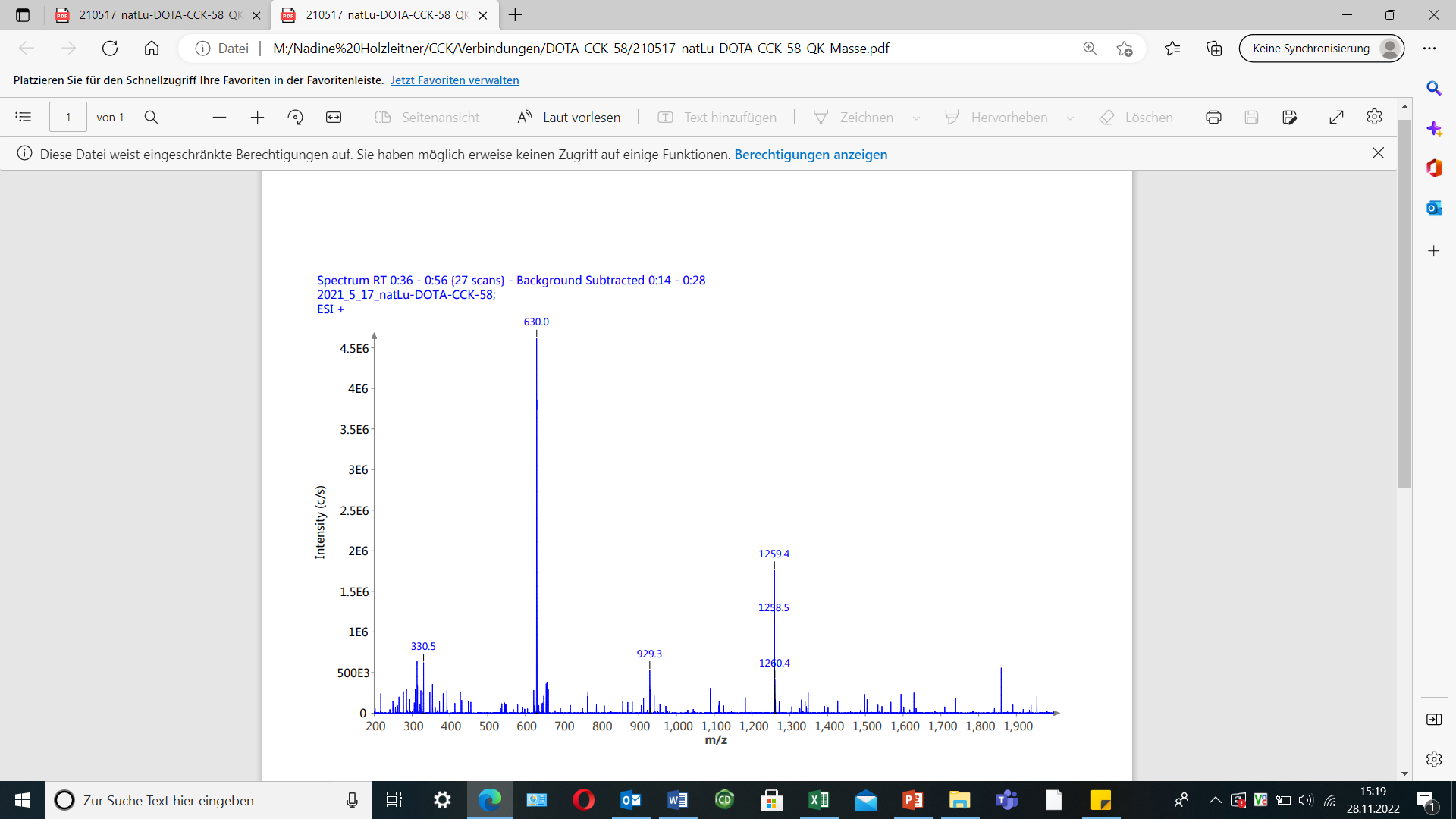


**Figure S8.** Confirmation of peptide identity and integrity for (**a**) [^nat^Lu]Lu-DOTA-CCK-62 and (**b**) [^177^Lu]Lu-DOTA-CCK-62 as analyzed by analytical (radio-)RP-HPLC (MultoKrom 100-5 C18, 5 μm, 125 × 4.6 mm, CS Chromatographie GmbH, Langerwehe, Germany; 10→70% MeCN in H_2_O + 0.1% TFA in 15 min). (**c**) Mass spectrum of [^nat^Lu]Lu-DOTA-CCK-62.

***[^nat^Lu]Lu-DOTA-CCK-62***: RP-HPLC (10→70% MeCN in H_2_O with 0.1% TFA, 15 min, λ = 220 nm): *t*_R_ = 11.0 min, K’ = 5.52; MS (ESI, positive): m/z calculated for C_53_H_68_LuN_11_O_14_: 1258.2, found: m/z = 1259.4 [M+H]^+^, 630.0 [M+2H]^2+^.

(a)

(b)

(c)


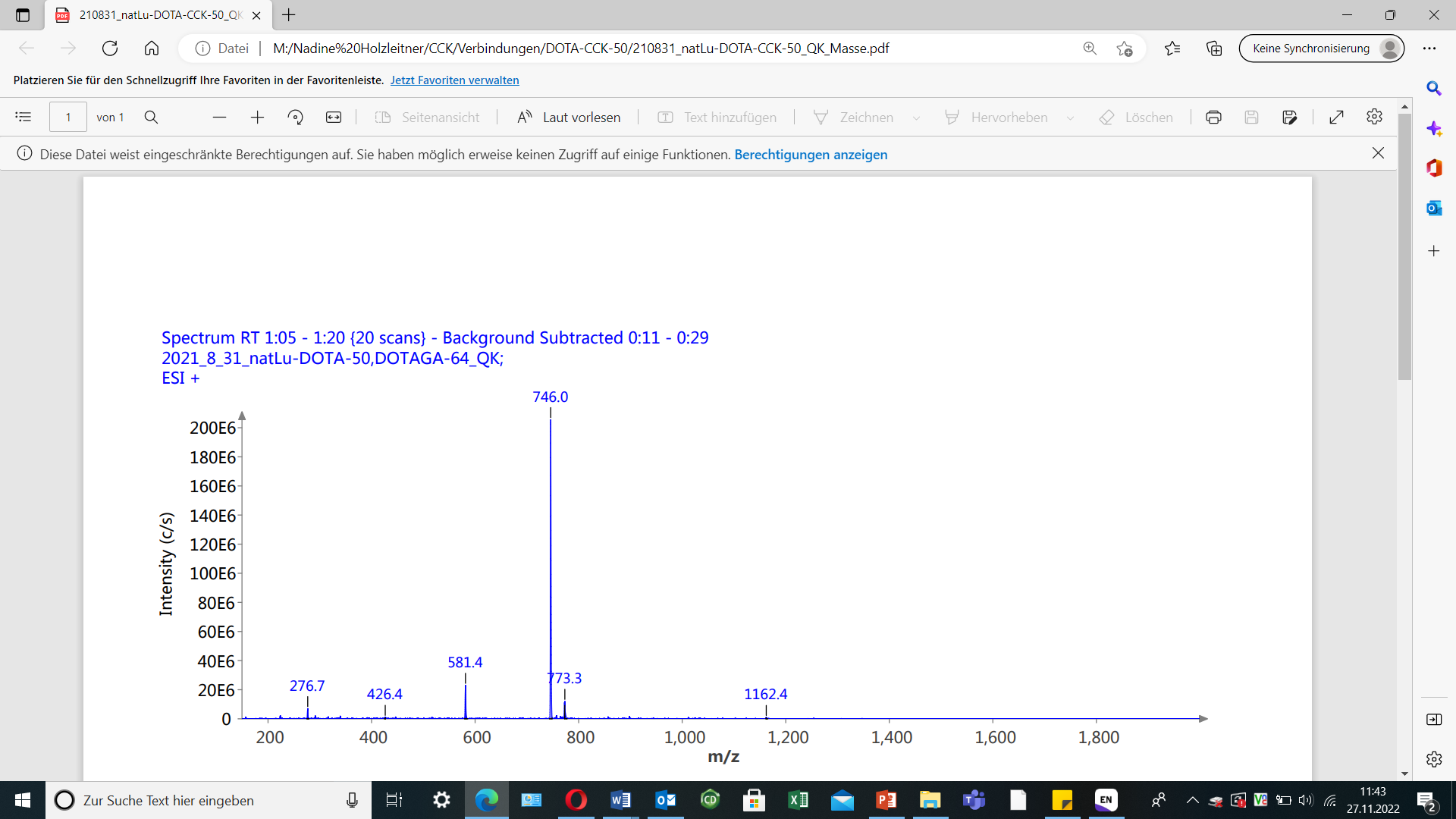


**Figure S9.** Confirmation of peptide identity and integrity for (**a**) [^nat^Lu]Lu-DOTA-CCK-63 and (**b**) [^177^Lu]Lu-DOTA-CCK-63 as analyzed by analytical (radio-)RP-HPLC (MultoKrom 100-5 C18, 5 μm, 125 × 4.6 mm, CS Chromatographie GmbH, Langerwehe, Germany; 10→70% MeCN in H_2_O + 0.1% TFA in 15 min). (**c**) Mass spectrum of [^nat^Lu]Lu-DOTA-CCK-63.

***[^nat^Lu]Lu-DOTA-CCK-63***: RP-HPLC (10→70% MeCN in H_2_O with 0.1% TFA, 15 min, λ = 220 nm): *t*_R_ = 11.3 min, K’ = 5.70; MS (ESI, positive): m/z calculated for C_64_H_90_LuN_11_O_19_: 1491.6, found: m/z = 746.0 [M+2H]^2+^.

**
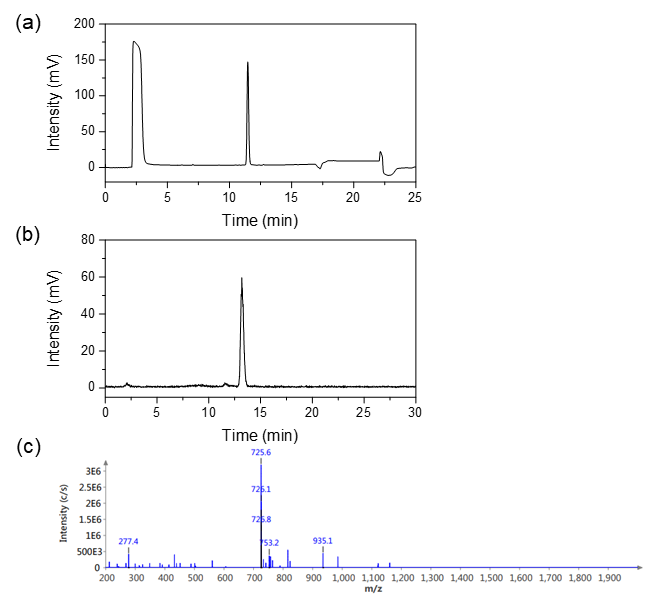
**

**Figure S10.** Confirmation of peptide identity and integrity for (**a**) [^nat^Lu]Lu-DOTA-CCK-64 as analyzed by analytical RP-HPLC (MultoKrom 100-5 C18, 5 μm, 125 × 4.6 mm, CS Chromatographie GmbH, Langerwehe, Germany; 10→70% MeCN in H_2_O + 0.1% TFA in 15 min) and (**b**) [^177^Lu]Lu-DOTA-CCK-64 as analyzed by analytical radio-RP-HPLC (10→30% MeCN in H_2_O + 0.1% TFA in 5 min; 30→60% MeCN in H_2_O + 0.1% TFA in 15 min). (**c**) Mass spectrum of [^nat^Lu]Lu-DOTA-CCK-64.

***[^nat^Lu]Lu-DOTA-CCK-64***: RP-HPLC (10→70% MeCN in H_2_O with 0.1% TFA, 15 min, λ = 220 nm): *t*_R_ = 11.5 min, K’ = 5.80; MS (ESI, positive): m/z calculated for C_62_H_86_LuN_11_O_18_: 1448.4, found: m/z = 725.6 [M+2H]^2+^.

**Analytical data of tetrapeptidic sequences**

(a)

(b)


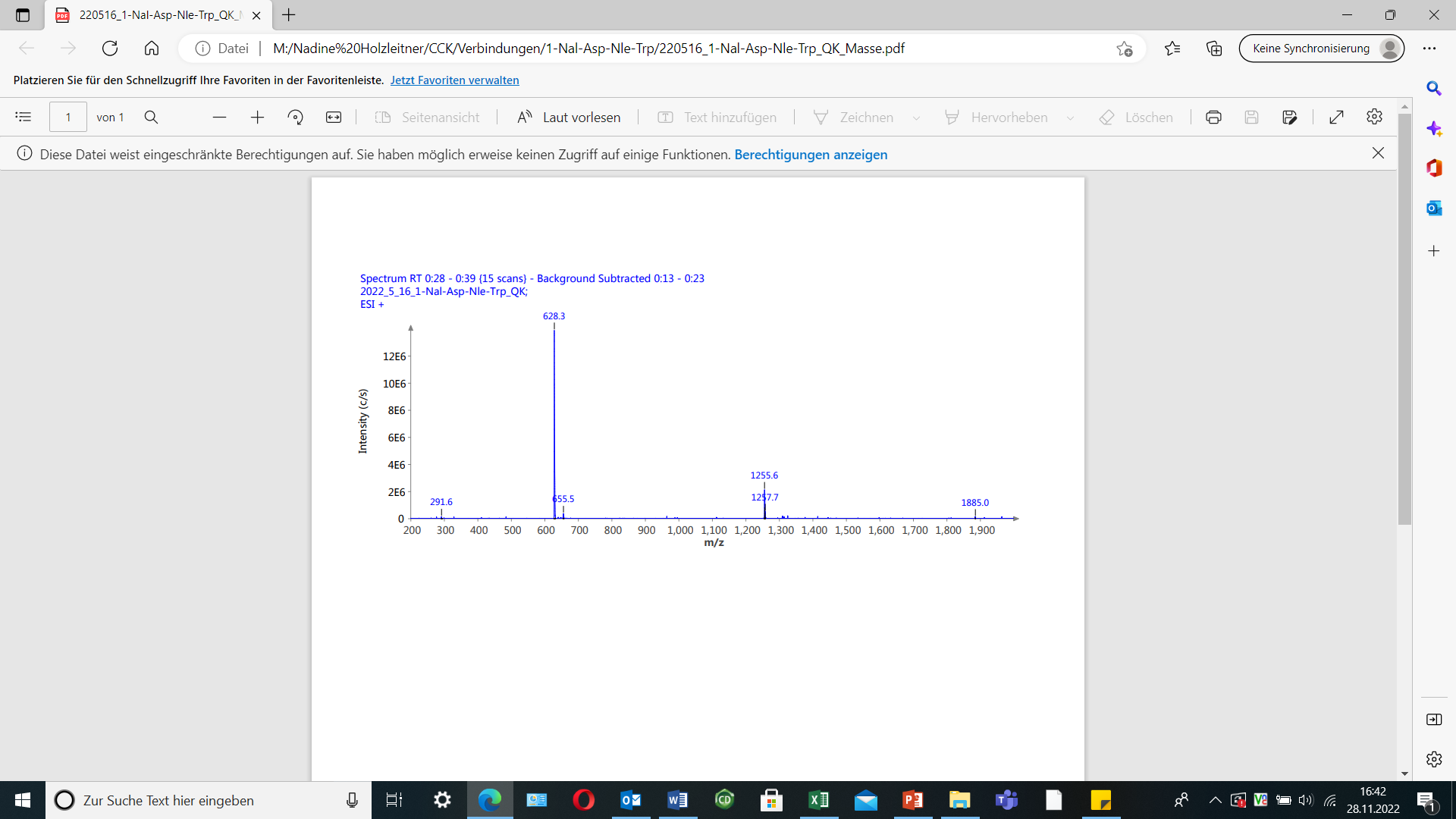


**Figure S11.** Confirmation of peptide identity and integrity for (**a**) *H*-Trp-Nle-Asp-1-Nal-NH_2_ as analyzed by analytical RP-HPLC (MultoKrom 100-5 C18, 5 μm, 125 × 4.6 mm, CS Chromatographie GmbH, Langerwehe, Germany; 10→70% MeCN in H_2_O + 0.1% TFA in 15 min). (**b**) Mass spectrum of *H*-Trp-Nle-Asp-1-Nal-NH_2_.

***H*-Trp-Nle-Asp-1-Nal-NH_2_ (B1):** RP-HPLC (10→70% MeCN in H_2_O with 0.1% TFA, 15 min, λ = 220 nm): *t*_R_ = 10.6 min, K’ = 5.28; MS (ESI, positive): m/z calculated for C_34_H_40_N_6_O_6_: 628.3, found: m/z = 1255.6 [2M+H]^+^, 628.3 [M+H]^+^.

(a)

(b)


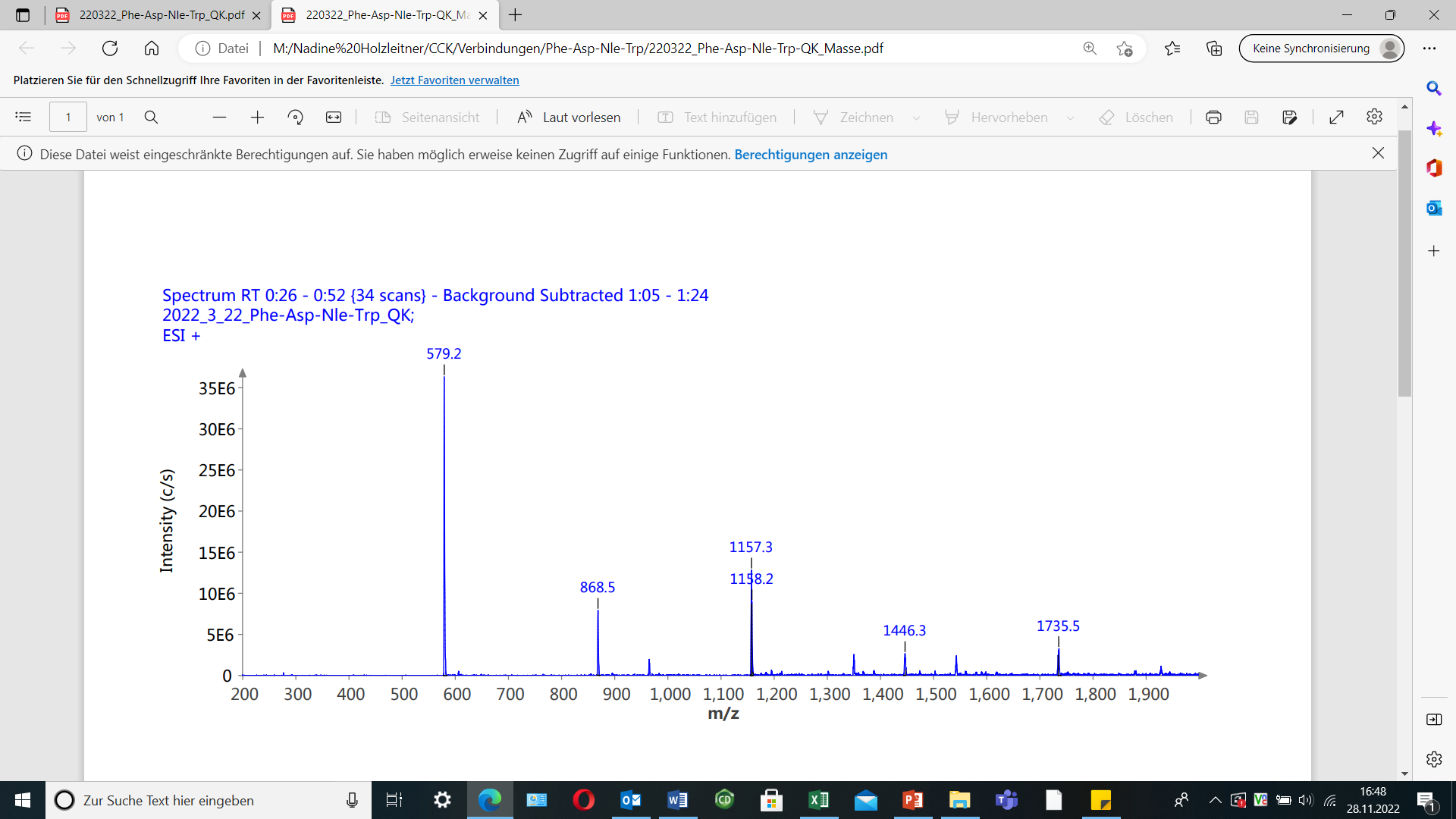


**Figure S12.** Confirmation of peptide identity and integrity for (**a**) *H-*Trp-Nle-Asp-Phe-NH_2_ as analyzed by analytical RP-HPLC (MultoKrom 100-5 C18, 5 μm, 125 × 4.6 mm, CS Chromatographie GmbH, Langerwehe, Germany; 10→70% MeCN in H_2_O + 0.1% TFA in 15 min). (**b**) Mass spectrum of *H*-Trp-Nle-Asp-Phe-NH_2_.

***H*-Trp-Nle-Asp-Phe-NH_2_ (B2):** RP-HPLC (10→70% MeCN in H_2_O with 0.1% TFA, 15 min, λ = 220 nm): *t*_R_ = 9.2 min, K’ = 4.45; MS (ESI, positive): m/z calculated for C_30_H_38_N_6_O_6_: 578.3, found: m/z = 1157.3 [2M+H]^+^, 579.2 [M+H]^+^.

(a)

(b)


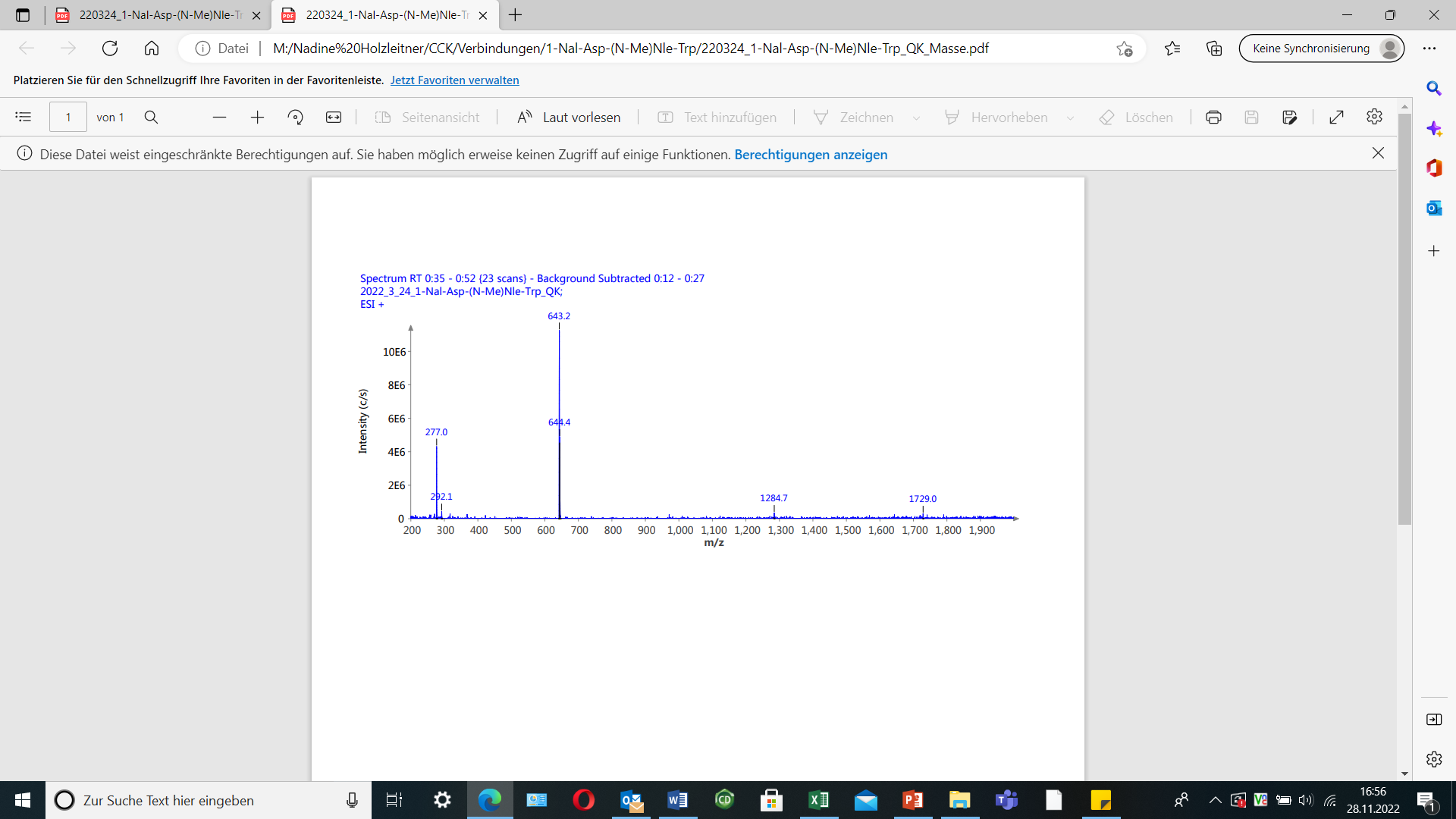


**Figure S13.** Confirmation of peptide identity and integrity for (**a**) *H-*Trp-(*N*-Me)Nle-Asp-1-Nal-NH_2_ as analyzed by analytical RP-HPLC (MultoKrom 100-5 C18, 5 μm, 125 × 4.6 mm, CS Chromatographie GmbH, Langerwehe, Germany; 10→70% MeCN in H_2_O + 0.1% TFA in 15 min). (**b**) Mass spectrum of *H*-Trp-(*N*-Me)Nle-Asp-1-Nal-NH_2_.

***H*-Trp-(*N*-Me)Nle-Asp-1-Nal-NH_2_ (B3):** RP-HPLC (10→70% MeCN in H_2_O with 0.1% TFA, 15 min, λ = 220 nm): *t*_R_ = 11.5 min, K’ = 5.82; MS (ESI, positive): m/z calculated for C_35_H_42_N_6_O_6_: 642.3, found: m/z = 1284.7 [2M+H]^+^, 643.2 [M+H]^+^.

(a)

(b)


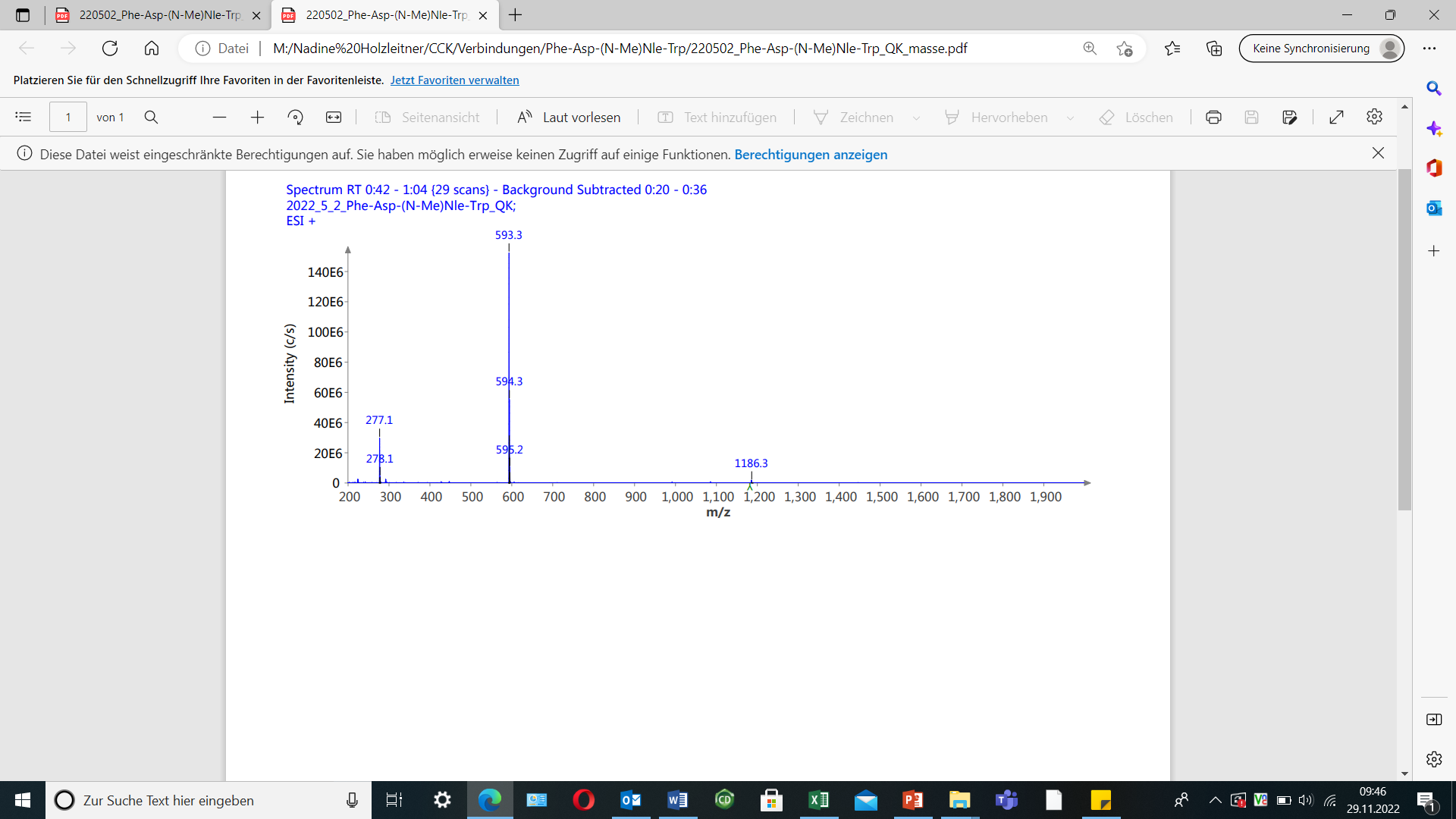


**Figure S14.** Confirmation of peptide identity and integrity for (**a**) *H-*Trp-(*N*-Me)Nle-Asp-Phe-NH_2_ as analyzed by analytical RP-HPLC (MultoKrom 100-5 C18, 5 μm, 125 × 4.6 mm, CS Chromatographie GmbH, Langerwehe, Germany; 10→70% MeCN in H_2_O + 0.1% TFA in 15 min). (**b**) Mass spectrum of *H-*Trp-(*N*-Me)Nle-Asp-Phe-NH_2_.

***H*-Trp-(*N*-Me)Nle-Asp-Phe-NH_2_ (B4):** RP-HPLC (10→70% MeCN in H_2_O with 0.1% TFA, 15 min, λ = 220 nm): *t*_R_ = 10.3 min, K’ = 5.11; MS (ESI, positive): m/z calculated for C_31_H_40_N_6_O_6_: 592.3, found: m/z = 1186.3 [2M+H]^+^, 593.3 [M+H]^+^.

(a)

(b)


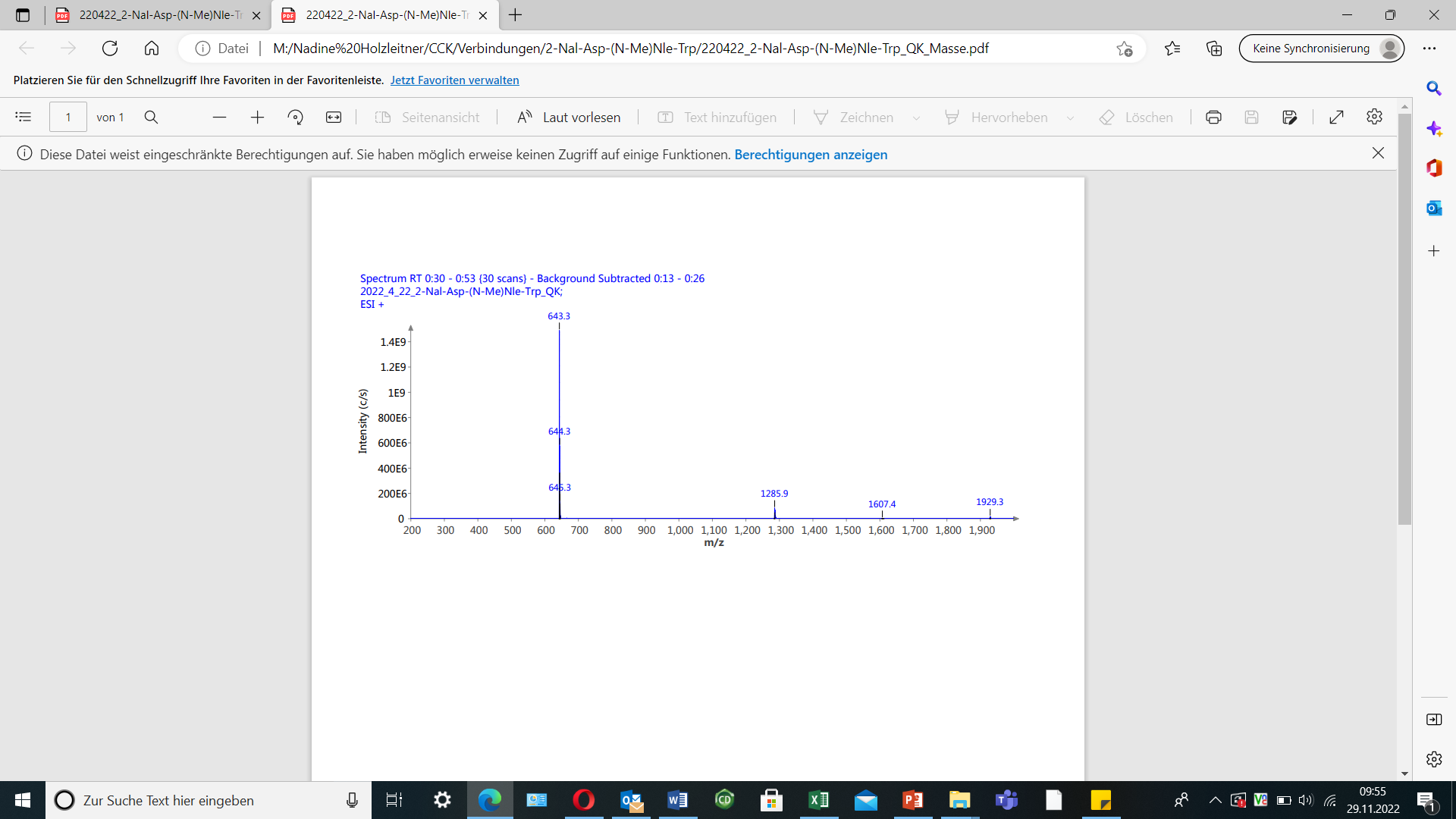


**Figure S15.** Confirmation of peptide identity and integrity for (**a**) *H-*Trp-(*N*-Me)Nle-Asp-2-Nal-NH_2_ as analyzed by analytical RP-HPLC (MultoKrom 100-5 C18, 5 μm, 125 × 4.6 mm, CS Chromatographie GmbH, Langerwehe, Germany; 10→70% MeCN in H_2_O + 0.1% TFA in 15 min). (**b**) Mass spectrum of *H*-Trp-(*N*-Me)Nle-Asp-2-Nal-NH_2_.

***H*-Trp-(*N*-Me)Nle-Asp-2-Nal-NH_2_ (B5):** RP-HPLC (10→70% MeCN in H_2_O with 0.1% TFA, 15 min, λ = 220 nm): *t*_R_ = 11.6 min, K’ = 5.88; MS (ESI, positive): m/z calculated for C_35_H_42_N_6_O_6_: 642.3, found: m/z = 1285.9 [2M+H]^+^, 643.3 [M+H]^+^.

(a)

(b)


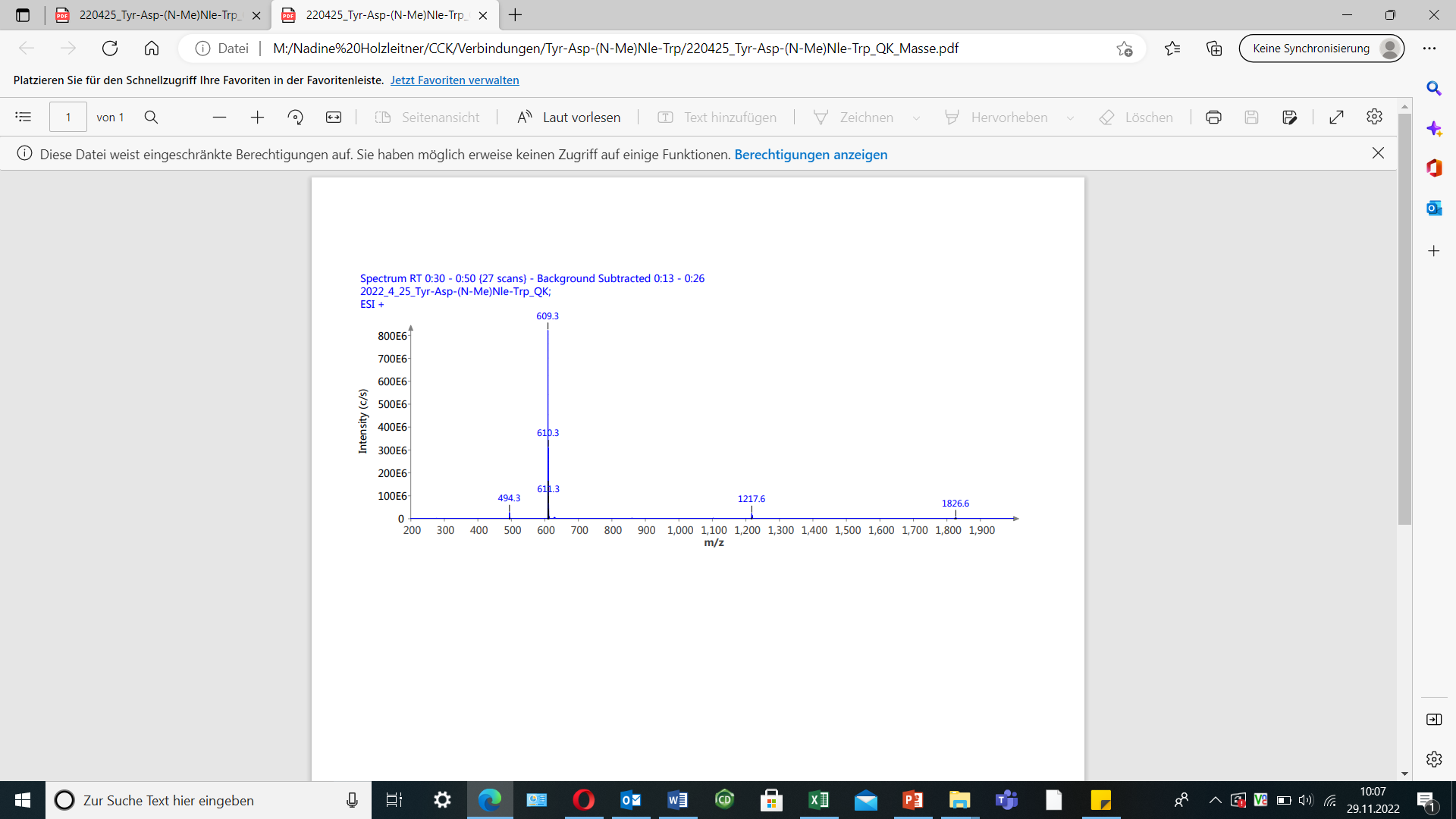


**Figure S16.** Confirmation of peptide identity and integrity for (**a**) *H-*Trp-(*N*-Me)Nle-Asp-1-Tyr-NH_2_ as analyzed by analytical RP-HPLC (MultoKrom 100-5 C18, 5 μm, 125 × 4.6 mm, CS Chromatographie GmbH, Langerwehe, Germany; 10→70% MeCN in H_2_O + 0.1% TFA in 15 min). (**b**) Mass spectrum of *H*-Trp-(*N*-Me)Nle-Asp-1-Tyr-NH_2_.

***H*-Trp-(*N*-Me)Nle-Asp-1-Tyr-NH_2_ (B6):** RP-HPLC (10→70% MeCN in H_2_O with 0.1% TFA, 15 min, λ = 220 nm): *t*_R_ = 8.8 min, K’ = 4.21; MS (ESI, positive): m/z calculated for C_31_H_40_N_6_O_7_: 608.3, found: m/z = 1217.6 [2M+H]^+^, 609.3 [M+H]^+^.

(a)

(b)


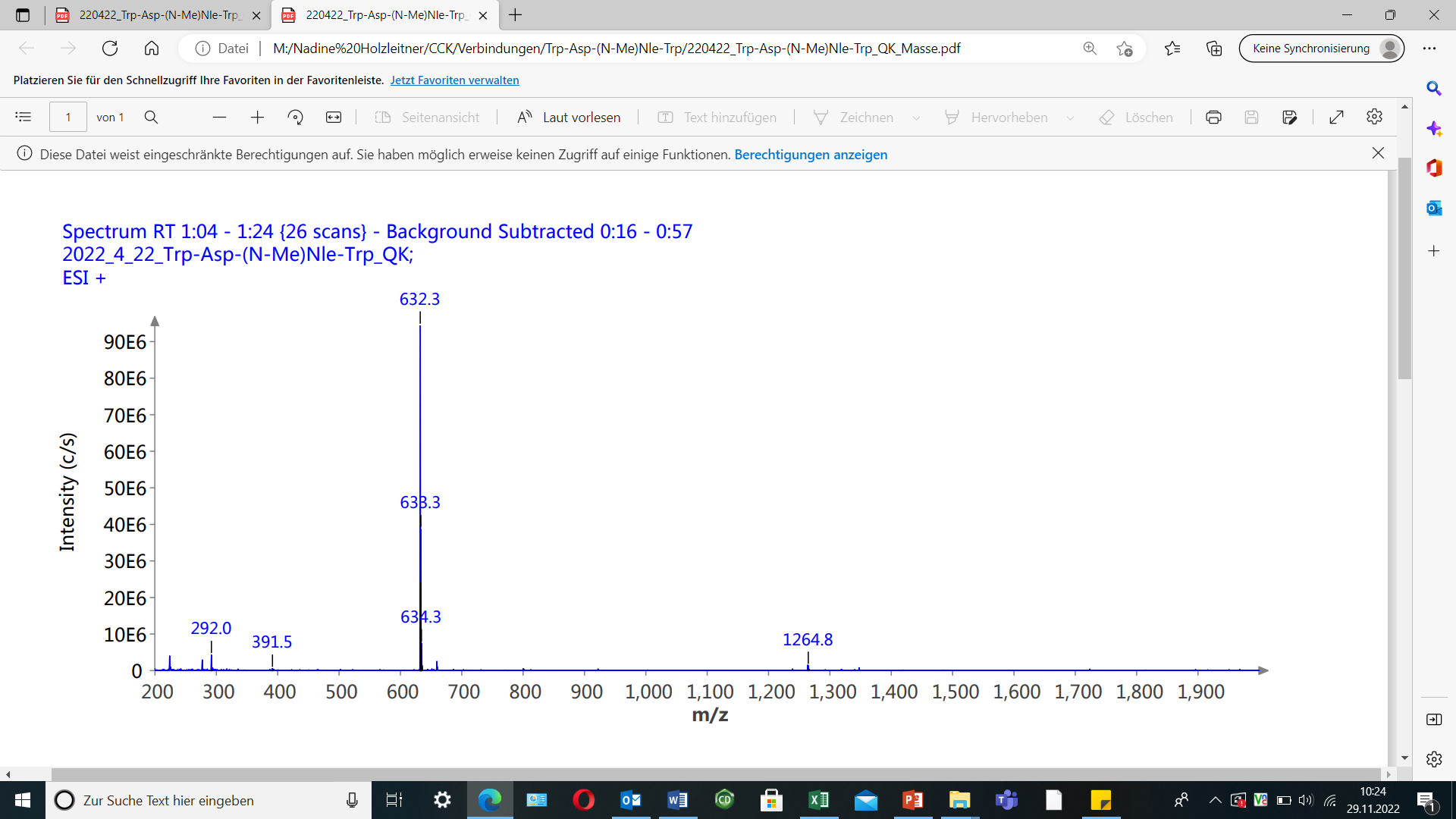


**Figure S17.** Confirmation of peptide identity and integrity for (**a**) *H-*Trp-(*N*-Me)Nle-Asp-Trp-NH_2_ as analyzed by analytical RP-HPLC (MultoKrom 100-5 C18, 5 μm, 125 × 4.6 mm, CS Chromatographie GmbH, Langerwehe, Germany; 10→70% MeCN in H_2_O + 0.1% TFA in 15 min). (**b**) Mass spectrum of *H*-Trp-(*N*-Me)Nle-Asp-Trp-NH_2_.

***H*-Trp-(*N*-Me)Nle-Asp-Trp-NH_2_ (B7):** RP-HPLC (10→70% MeCN in H_2_O with 0.1% TFA, 15 min, λ = 220 nm): *t*_R_ = 10.3 min, K’ = 5.11; MS (ESI, positive): m/z calculated for C_33_H_41_N_7_O_6_: 631.3, found: m/z = 1264.8 [2M+H]^+^, 632.3 [M+H]^+^.

**^177^Lu-Labeling**

For ^177^Lu-labeling experiments, [^177^Lu]LuCl_3_ dissolved in hydrochloric acid (0.04 M, 40 GBq/mL) was acquired from ITM Isotope Technologies Munich SE (Garching, Germany). Radiolabeling of the peptide precursor (1 nmol) was performed at 90°C for 15 min in a NaOAc-buffered (1 M, pH = 5.5) hydrochloric acid (0.04 M) solution. After radiolabeling, a sodium ascorbate (1 M in H_2_O) was added to prevent radiolysis and radiochemical purity was determined via radio-RP-HPLC and radio-TLC (instant thin layer chromatography paper impregnated with silica gel (iTLC-SG, Agilent Technologies Inc., Folsom, CA, United States); sodium citrate × 1.5 H_2_O (0.1 M)).

**Table S1.** CCK-2R affinity and lipophilicity data of the compounds evaluated. Affinity data were determined on AR42J cells (2.0 x 10^5^ cells/well/mL) and [^177^Lu]Lu-DOTA-PP-F11N (0.3 pmol/well) as radiolabeled reference (3 h, 37°C, RPMI 1640, 5 mM L-Gln, 5 mL non-essential amino acids (100x), 10% FCS + 5% BSA (*v*/*v*)).

| **Peptide** | ***IC*_50_** [nm] | **log*D*_7.4_** |
| --- | --- | --- |
| [^nat/177^Lu]Lu-DOTA-CCK-55 | 8.51 ± 1.16 | −2.09 ± 0.07 |
| [^nat/177^Lu]Lu-DOTA-CCK-56 | 4.92 ± 0.79 | −1.38 ± 0.07 |
| [^nat/177^Lu]Lu-DOTA-CCK-57 | 9.67 ± 1.74 | −1.94 ± 0.06 |
| [^nat/177^Lu]Lu-DOTA-CCK-58 | 8.21 ± 1.33 | −2.66 ± 0.06 |
| [^nat/177^Lu]Lu-DOTA-CCK-59 | 5.61 ± 0.48 | −1.26 ± 0.08 |
| [^nat/177^Lu]Lu-DOTA-CCK-60 | 4.16 ± 0.47 | −2.63 ± 0.05 |
| [^nat/177^Lu]Lu-DOTA-CCK-61 | 7.85 ± 0.43 | −2.56 ± 0.09 |
| [^nat/177^Lu]Lu-DOTA-CCK-62 | 98.9 ± 8.4 | −1.34 ± 0.08 |
| [^nat/177^Lu]Lu-DOTA-CCK-63 | 8.84 ± 1.25 | −2.13 ± 0.05 |
| [^nat/177^Lu]Lu-DOTA-CCK-64 | 7.64 ± 0.86 | −2.21 ± 0.07 |
| [^nat/177^Lu]Lu-DOTA-γ-MGS5 | 4.90 ± 0.78 | −2.24 ± 0.04 |
| *H*-Trp-Nle-Asp-1-Nal-NH_2_ (B1) | 297 ± 15 | not determined (n.d.) |
| *H*-Trp-Nle-Asp-Phe-NH_2_ (B2) | 466 ± 88 | n.d. |
| *H*-Trp-(*N*-Me)Nle-Asp-1-Nal-NH_2_ (B3) | 5.88 ± 0.52 | n.d. |
| *H*-Trp-(*N*-Me)Nle-Asp-Phe-NH_2_ (B4) | 4.49 ± 0.56 | n.d. |
| *H*-Trp-(*N*-Me)Nle-Asp-2-Nal-NH_2_ (B5) | 102 ± 9 | n.d. |
| *H*-Trp-(*N*-Me)Nle-Asp-Tyr-NH_2_ (B6) | 5.70 ± 0.48 | n.d. |
| *H*-Trp-(*N*-Me)Nle-Asp-Trp-NH_2_ (B7) | 18.8 ± 0.5 | n.d. |

**Table S2.** *In vitro* stability of [^177^Lu]Lu-DOTA-CCK-55, [^177^Lu]Lu-DOTA-CCK-62, [^177^Lu]Lu-DOTA-CCK-63 and [^177^Lu]Lu-DOTA-γ-MGS5 after incubation for 24 h at 37°C in human serum. Values are depicted in percent of intact peptide as analyzed by radio-RP-HPLC (MultoKrom 100-5 C18, 5 μm, 125 × 4.6 mm, CS Chromatographie GmbH, Langerwehe, Germany; 10→30% MeCN in H_2_O + 0.1% TFA in 5 min; 30→60% MeCN in H_2_O + 0.1% TFA in 5 min).

| **Compound** | Intact Peptide (%) |
| --- | --- |
| [^177^Lu]Lu-DOTA-CCK-55 | 87.5 ± 1.9 |
| [^177^Lu]Lu-DOTA-CCK-62 | 97.9 ± 1.8 |
| [^177^Lu]Lu-DOTA-CCK-63 | 57.4 ± 3.7 |
| [^177^Lu]Lu-DOTA-CCK-64 | 43.8 ± 2.3 |
| [^177^Lu]Lu-DOTA-γ-MGS5 | 96.8 ± 2.8 |
